# Lung cancer cells survive epidermal growth factor receptor tyrosine kinase inhibitor exposure through upregulation of cholesterol synthesis

**DOI:** 10.1096/fba.2019-00081

**Published:** 2019-12-28

**Authors:** Mark C. Howell, Ryan Green, Roukiah Khalil, Elspeth Foran, Waise Quarni, Rajesh Nair, Stanley Stevens, Aleksandr Grinchuk, Andrew Hanna, Shyam Mohapatra, Subhra Mohapatra

**Affiliations:** ^1^ Molecular Medicine Department University of South Florida Tampa FL USA; ^2^ Center for Research & Education in Nanobioengineering University of South Florida Tampa FL USA; ^3^ Transgenex Nanobiotech Inc Tampa FL USA; ^4^ Cell Biology, Microbiology, and Molecular Biology College of Arts and Sciences University of South Florida Tampa FL USA; ^5^ Division of Translational Medicine Internal Medicine Morsani College of Medicine University of South Florida Tampa FL USA; ^6^ James A Haley Veterans Hospital Tampa FL USA

**Keywords:** cholesterol, drug tolerance, EGFR TKIs, lung cancer

## Abstract

Epidermal growth factor receptor (EGFR) tyrosine kinase inhibitors (TKIs) provide clinical benefits over chemotherapy for lung cancer patients with EGFR activating mutations. Despite initial clinical responses, long‐term efficacy is not possible because of acquired resistance to these therapies. We have developed EGFR TKI drug‐tolerant (DT) human lung cancer cell lines as a model for de novo resistance. Mass spectroscopic analysis revealed that the cytochrome P450 protein, CYP51A1 (Lanosterol 14α‐demethylase), which is directly involved with cholesterol synthesis, was significantly upregulated in the DT cells. Total cellular cholesterol, and more specifically, mitochondrial cholesterol, were found to be upregulated in DT cells. We then used the CYP51A1 inhibitor, ketoconazole, to downregulate cholesterol synthesis. In both parental and DT cells, ketoconazole and EGFR TKIs acted synergistically to induce apoptosis and overcome the development of EGFR tolerance. Lastly, this combination therapy was shown to shrink the growth of tumors in an in vivo mouse model of EGFR TKI resistance. Thus, our study demonstrates for the first time that ketoconazole treatment inhibits upregulation of mitochondrial cholesterol and thereby overcomes EGFR‐TKI resistance in lung cancer cells.

**Abbreviations**
Akt: Serine‐threonine protein kinase AKT1
ANOVA: Analysis of variance
Bad: BCL2 associated agonist of cell death
Bak: Bcl‐2 homologous antagonist killer
Bax: Bcl‐2‐associated X protein
Bcl‐2: B‐cell lymphoma 2
Bcl‐xL: B‐cell lymphoma extra‐large
Bid: BH3 Interacting Domain Death Agonist
Bim: Bcl‐2‐like protein 11
CO2: Carbon Dioxide
COX4: Cytochrome c oxidase subunit 4
CYP51A1: Lanosterol 14α‐demethylase
DHCR24: 24‐Dehydrocholesterol reductase
DHCR7: 7‐Dehydrocholesterol reductase
DMSO: Dimethyl sulfoxide
DT: Drug-tolerant
Ebp: Delta(8)‐Delta(7) sterol isomerase
EGF: Epidermal growth factor
EGFR: Epidermal growth factor Receptor
Erk: Extracellular signal‐regulated kinases
FBS: Feta Bovine Serum
FGFR: Fibroblast growth factor receptors
FiSS: Fiber inspired smart scaffold
HER2: Human epidermal growth factor receptor 2
HMG‐CoA: β‐Hydroxy β‐methylglutaryl‐CoA
HPRT: Hypoxanthine‐guanine phosphoribosyltransferase
IC50: Half maximal inhibitory concentration
ITRAQ: Isobaric tag for relative and absolute quantitation
JAK: Janus kinases
LDL: Low‐density lipoprotein
LLC: Lewis lung carcinoma
LSS: Lanosterol Synthase
LXRs: liver X receptors
Mapk: Mitogen‐activated protein kinase
MBCD: Methyl‐β‐cyclodextrin
Mcl‐1: Induced myeloid leukemia cell differentiation protein
Mek: Mitogen‐activated protein kinase kinase
MET: c‐Met proto‐oncogene protein
MOMP: Mitochondrial outer membrane permeabilization
mTor: Mammalian target of rapamycin
mTorc2: Mammalian target of rapamycin complex 2
NFκB: nuclear factor kappa‐light‐chain‐enhancer of activated B cells
Noxa: Phorbol‐12‐myristate‐13‐acetate‐induced protein 1
NSCLC: Non‐small‐cell lung carcinoma
PARP: Poly ADP ribose polymerase
PBS: Phosphate buffered saline
PI: Propidium iodide
PI3K: Phosphoinositide 3‐kinase
PIK3CA: Phosphatidylinositol‐4,5‐bisphosphate 3‐kinase, catalytic subunit alpha
Puma: p53 upregulated modulator of apoptosis
Raf: Rapidly Accelerated Fibrosarcoma kinase
Ras: p21/Ras family small GTPase
SC5D: Lathosterol oxidase
SEM: Standard error of the mean
SOAT1: Sterol O‐acyltransferase
Src: Proto‐oncogene tyrosine‐protein kinase Src
SREBPs: Sterol regulatory element‐binding proteins
Stat3: Signal transducer and activator of transcription 3
TKI: Tyrosine kinase inhibitor
VEGFR: Vascular endothelial growth factor receptor
Wnt: Proto‐Oncogene Wnt‐1

## 1 INTRODUCTION

About 20% of all non‐small cell lung cancer (NSCLC) patients harbor an epidermal growth factor receptor (EGFR) activating mutation.^1^ EGFR tyrosine kinase inhibitors (EGFR‐TKIs) have been shown to provide clinical benefits over chemotherapy for lung cancer patients with EGFR activating mutations.^2^ Some first generation‐(gefitinib, erlotinib, lapatinib), second generation‐(afatinib), and third‐generation (osimertinib) EGFR TKIs are clinically approved to treat NSCLC patients.^3^ Lapatinib is a special case, as it is qualified as a dual TKI, which interrupts both the HER2 and EGFR pathways, and is commonly used to treat patients with metastatic breast cancer whose tumors overexpress HER2.^4^ Despite the initial clinical responses to EGFR targeted therapies, acquired drug resistance hampers TKI effectiveness in most patients.^1^, ^3^ Target alteration, increased ligand production, increased downstream pathway activation, and alternative pathway activation have all been proposed as mechanisms of resistance to EGFR TKIs.^1^, ^3^ Numerous cellular signaling pathways have been implicated in EGFR TKI resistance.^1^, ^5^, ^6^, ^7^, ^8^, ^9^, ^10^, ^11^, ^12^, ^13^, ^14^, ^15^, ^16^, ^17^

It has been shown that statins, which work to lower cholesterol, in combination with EGFR TKIs provide additional benefits over EGFR TKIs alone. A population‐based case‐control study, including 1707 statin and 6828 non‐statin matched lung cancer cohorts with EGFR TKI treatment, found that statin use was associated with a reduced risk of death, a significantly longer median progression‐free survival, and significantly longer median overall survival.^18^ It has been found that a combination treatment of EGFR TKIs and simvastatin is able to overcome T790M mediated EGFR TKI resistance through downregulation of AKT/β‐catenin survival signaling.^16^ Simvastatin treatment was shown to be able to restore expression of proapoptotic protein, BIM and induce apoptotic cell death in H1975 cells which harbor the T790M EGFR mutation.^17^ Another study suggested that a combination of lovastatin and gefitinib can overcome resistance to gefitinib through downregulation of RAS and inhibition of RAF/ERK and AKT.^19^ Two studies have found that lovastatin induced cholesterol depletion from lipid rafts and was able to restore sensitivity to gefitinib in resistant cell lines.^20^, ^21^ Taken together, these studies highlight the potential for a combination therapy targeting cholesterol synthesis along with EGFR inhibition.

The lipid cholesterol, an essential component of plasma membranes and lipid rafts, plays important roles in maintaining cellular homeostasis via intracellular signal transduction.^22^, ^23^ Lipid rafts are small domains within the cell membrane that are less fluid than the neighboring membrane due to the fact that they are enriched in cholesterol and sphingolipids. EGFR has been shown in multiple studies to be associated with lipid rafts.^24^, ^25^, ^26^ In the case of EGFR TKI activity, a few studies have been done to determine the role of lipid rafts in the cellular responses to EGFR inhibition by TKIs.^20^, ^21^ One study found that cholesterol levels in lipid rafts from gefitinib resistant NSCLC cell lines were significantly higher than those from a gefitinib sensitive cell line.^20^ Another study found that EGFR localized to cell membrane lipid rafts in EGFR TKI resistant cell lines and that the lipid rafts were providing a platform for activation of Akt signaling even in the absence of EGFR kinase activity.^21^

Another potential role for cholesterol modulation of the EGFR pathway lies in the mitochondrial membrane and its role in apoptosis. Human cells have two major apoptosis signaling pathways: the extrinsic or death receptor pathway and the intrinsic or mitochondrial pathway.^27^, ^28^ The intrinsic pathway exerts its apoptotic effects via mitochondrial outer membrane permeabilization (MOMP), the release of cytochrome c, and activation of the caspase cascade.^27^, ^28^ For MOMP to occur, a number of Bcl‐2 family proteins must be engaged.^27^, ^28^ EGFR TKIs have been shown to mediate their apoptotic effects via the intrinsic pathway of apoptosis through inhibition of Akt and Erk dependent pathways that cause changes in the expression level of Bcl‐2 family members and allow for activation of apoptosis.^27^, ^28^ In some cancers, enriched mitochondrial cholesterol levels have been shown to induce resistance to apoptotic signals.^29^, ^30^, ^31^ Several studies have shown that elevated levels of mitochondrial cholesterol in cancer cells may be able to protect against mitochondrial apoptosis by changing mitochondrial membrane dynamics and permeability.^29^, ^30^, ^31^ In these cases, a cholesterol‐mediated decrease in mitochondrial membrane fluidity reduces the ability of Bax to insert into the mitochondrial membrane which impairs MOMP, and the release of cytochrome c in response to Bax.^29^, ^30^, ^31^ So far, however, no studies have linked this phenomenon to EGFR TKI exposure and resistance.

The initial mechanisms that promote survival of the drug‐tolerant (DT) cells may be sufficient to prevent apoptosis but may not fully recapitulate the oncogenic signaling provided by EGFR. These cells provide a reservoir from which genetic mechanisms of acquired resistance can evolve.^32^, ^33^, ^34^ We hypothesize that a better understanding of cholesterol's role in the mechanisms responsible for initial cell survival after exposure to EGFR TKIs will allow us to design a therapy that is able to target these DT cells and prevent the development of drug resistance.

## 2 MATERIALS AND METHODS

This section presents the general molecular biology techniques used during the study.

### 2.1 In vitro studies

#### 2.1.1 Amplex Red cholesterol assay

Cholesterol was extracted using a 30:20 solution of Hexane/Isopropanol. Cholesterol content was determined using the Amplex Red Cholesterol Assay Kit (Thermo Fisher Scientific, ) according to the manufacturer's protocol. The assay detects both free cholesterol and cholesteryl esters. Cholesterol content was normalized to cell count and graphed as fold change over control.

#### 2.1.2 Cell culture‐ monolayer and scaffold

Cell lines were purchased from the American type culture collection (ATCC) and passaged no more than 25 times. Cells were cultured in a humidified incubator at 37C in a 5% CO_2_ (Carbon Dioxide) atmosphere. Cells were cultured in tissue culture treated plates in the appropriate complete cell culture media [DMEM or RPMI (GE Healthcare) containing 10% fetal bovine serum (FBS) (Atlanta Biologicals) and 1% penicillin/streptomycin (GE Healthcare)]. The Fiber Inspired Smart Scaffold (FiSS) was prepared and seeded with cells as previously described.^35^, ^36^, ^37^ Tumor derived biopsy cultures: mice were injected subcutaneously on flanks with 3 million H1650 parental (right) and DT (left) cells. The tumors were harvested when they reached about 10mm in diameter and digested using MACS Miltenyi Biotec Mouse Tumor Dissociation kit and gentle MACS Dissociator (Miltenyi Biotec) according to the manufacturer's protocol. Tumor cell suspensions were then plated on the FiSS just as cell line suspensions.

#### 2.1.3 Cell Titer Glo IC50 assay

Cells were treated with varying concentrations of drug for 48 hours in monolayer (duplicates) and FiSS cultures (triplicates). Drugs were added on day 5 of FiSS culture or after 24 hours of monolayer culture. Cell viability was determined using Cell Titer Glo assay (Promega) according to the manufacturer's protocol. Luminescence was measured in a white well‐plate in a Bio‐Tek Synergy H4 plate reader (BioTek). Average luminescence value for each group was plotted as a percentage of the control group. A non‐linear regression was then run to calculate the IC50 value. Drugs were obtained from LC Laboratories and Sigma Aldrich. 2% Lipogro Bovine Cholesterol Concentrate was purchased from Rocky Mountain Biologicals, Inc.

#### 2.1.4 Combination index

Combination index was calculated using the formula 21:

$$\text{CI}=\left\{\frac{\left[\text{IC}50\;\text{LP}\left(\text{at X dose KC}\right)\right]}{\text{IC}50\;\text{LP}}\right\}+\left\{\frac{\left[\text{X dose KC}\right]}{\text{IC50 KC}}\right\}$$

A CI < 1 = synergy, a CI = 1 = additive effect, and a CI > 1 = an antagonistic effect.

#### 2.1.5 DT cell line generation

Lung cancer cell lines containing EGFR mutations (H1650 and H1975) in addition to EGFR wild type cell lines (H1299 and LLC1) were used to represent a range of NSCLCs (Table 1). H1975 cells have stable expression of luciferase and green fluorescent protein (GFP) and LLC cells have stable expression of luciferase. These two cell lines were purchased from Genecopoeia (Maryland USA) (H1975) and PerkinElmer (LLC) and were grown according to the manufacturer's protocol. Cells were grown in appropriate complete cell culture media containing the highest static concentration of the respective EGFR TKI that they would proliferate in and used for experiments from day 21 to day 60.

**Table 1:** Lung cancer cell lines used in this study

Driver mutations							
CELL LINES	SPECIES	CDKN2A	EGFR	KRAS	NRAS	PIK3CA	TP53
H1650	Human	c.1_471del471	c.2235_2249del15	WT	WT	WT	c.673‐2A > G
H1975	Human	c.205G > T	c.2369C > T c.2573T > G	WT	WT	c.353G > A	c.205G > T
H1299	Human	WT	Overexpress	WT	c.181C > A	WT	Deletion
LLC	Mouse	WT	Overexpress	p.G12Chet	p.Q61Hhet	WT	WT

#### 2.1.6 Flow cytometry

All flow cytometry experiments were performed using a Becton Dickenson Biosciences (BD) FACS Canto II system at the University of South Florida COM Fred Wright Jr Flow Cytometry Core. All analysis and graphing for flow cytometry experiments was done using FlowJo 8.7 software (BD).

##### Annexin V assay

Cells were treated with lapatinib and/or ketoconazole for 48 hours in monolayer cultures. They were then stained for Annexin V and PI using the FITC Annexin V Apoptosis Detection Kit I (BD Pharmingen) or the eBioscience™ Annexin V Apoptosis Detection Kit APC (Thermo Fisher Scientific) according to the manufacturer's protocol.

##### JC‐1 assay

Cells were treated with lapatinib and/or ketoconazole for 48 hours in monolayer cultures. They were stained with JC‐1 (Thermo Fisher Scientific) according to the manufacturer's protocol. Data was graphed as mean PE/Alexa 488 ratio.

#### 2.1.7 Lipid raft staining

Cells were treated with lapatinib for 48 hours in monolayer cultures. Lipid rafts were stained for using the Vybrant™ Alexa Fluor™ 555 Lipid Raft Labeling Kit (Thermo Fisher Scientific) according to the manufactures’ protocol. Z‐stack images (600×) were taken using the Olympus FLUOVIEW FV1000 confocal laser scanning microscope. Max projections of the confocal Z‐stack images (600×) were analyzed for fluorescence intensity per total cell area using ImageJ software (National Institute of Health). Data were graphed as fold change over control.

#### 2.1.8 Mass spectroscopy

Whole cell lysate for each cell line was run in duplicate. Peptides for each sample were labeled using ITRAQ labeling kit. Data were collected using the Q Exactive Plus mass spectrometer (Thermo Fisher Scientific) and analyzed first using MaxQuant proteomics software (Max‐Planck‐Institute of Biochemistry) and next uploaded into Scaffold software (Proteome Software) for statistical analysis. Identified proteins were first subjected to a Mann‐Whitney U test to look for significant differences in protein abundance. Protein abundance is measured by the average intensities between replicates. Proteins that were identified to have a *P*‐value of *P *≤ .05 were further analyzed to characterize the fold change difference between the groups.

#### 2.1.9 Mitochondrial isolation

Cell pellets were collected, and mitochondria were extracted using the Mitochondria Isolation Kit for Cultured Cells (Thermo Fisher Scientific) according to the manufacturer's protocol.

#### 2.1.10 Nuc blue fluorescent microscopy

Tumoroid formation was assessed using fluorescent microscopy (EVOS, Thermo Fisher Scientific) after nuclear staining with Nuc Blue dye (Thermo Fisher Scientific) according to the manufacturer's protocol.

#### 2.1.11 Quantitative reverse transcriptase PCR (qPCR)

Total cellular RNA was extracted from cell pellets using Trizol (Thermo Fisher Scientific) according to the manufacturer's protocol. RNA was quantified using the Nanodrop (Thermo Fisher Scientific). One microgram of RNA was then reverse transcribed using the Maxima cDNA Reverse Transcription Kit (Thermo Fisher Scientific) according to the manufacturer's protocol. qPCR performed on the cDNA was used to quantitate the relative expression levels of certain genes to hypoxanthine‐ guanine phosphoribosyltransferase (HPRT) or β‐actin as a control. Real time analysis was performed using BlazeTaq SYBR Green qPCR Mix 2.0 (Genecoepia), according to the manufacturer's protocol, in a Bio Rad CFX‐384 thermocycler using primers obtained from Integrated DNA Technologies (IDT). (See Table 2 for primer sequences). The data were analyzed using ΔCt and ΔΔCt calculations and expression of all genes was normalized to HPRT expression as a housekeeping gene. Average fold change ±SEM, compared to control, was then calculated. Data analysis was performed using the CFX Maestro software (Bio‐Rad).

**Table 2:** Human primer sequences used for quantitative real time PCR

Gene	Strand	Primer sequence
β‐Actin	Forward	CAAACATGATCTGGGTCATCTTCT
	Reverse	CAAACATGATCTGGGTCATCTTCT
DHCR7	Forward	ACATGCTCGGCTCTCGGAC
	Reverse	AGGTATAGAGCTGGGCGGCT
DHCR24	Forward	ATCGCAGCTTTGTGCGATG
	Reverse	CACCAGGAAACCCAGCGT
HPRT	Forward	GAAAGGGTGTTTATTCCTCATGG
	Reverse	CAGTGCTTTGATGTAATCCAGCAG
LSS	Forward	GGCAGACGTGGACCTACC
	Reverse	GAAAAGTGGGCCACCATAATC
SREBF2	Forward	CCCTTCAGTGCAACGGTCATTCAC
	Reverse	TGCCATTGGCCGTTTGTGTC

#### 2.1.12 Western immunoassay

##### WES automated capillary western blotting

Total protein was extracted from cell pellets using RIPA buffer (Thermo Fisher Scientific) according to the manufacturer's protocol. Protein concentration was determined using the Pierce Coomassie Protein Assay Kit (Thermo Fisher Scientific) according to the manufacturer's protocol. The expression of the indicated proteins was determined using the WES (Protein Simple) automated western blotting system according to the manufacturer's protocol. Total protein expression was analyzed using area under the curve (AUC) measurements generated using Compass software for Simple Western (Protein Simple). Housekeeping genes were used to quantify relative protein expression. Average AUC ± SEM as a percentage of control was calculated. Images were derived using Compass Software (Protein Simple). For antibodies used: see Table 3.

**Table 3:** Antibodies used in immunoblotting

Protein	Company	Catalog number
AKT	Cell signaling	4685
p‐AKT	Cell signaling	4060
α‐actinin	Santa Cruz biotechnology	Sc‐17829
β‐actin	Sigma‐Aldrich	A2228
Cleaved Caspase‐9	Cell signaling	9501P
Cox IV	Novus biologicals	NBP2‐43540
Cytochrome c	Novus biologicals	MAB897
CYP51A1	Sigma‐Aldrich	HPA041325
DHCR24	Abcam	Ab40490
EGFR	Cell signaling	4267
p‐EGFR	Cell signaling	3777
LSS	Abcam	Ab80364
ERK1/2	Cell signaling	4695
p‐ERK1/2	Cell signaling	4370
PARP1	Cell signaling	9542
SREBP2	Abcam	Ab30682
Survivin	Cell signaling	2808
Vinculin	Santa Cruz biotechnology	Sc‐25336
Anti‐Mouse	EMD millipore	2 854 655
Anti‐Rabbit	Santa Cruz biotechnology	Sc‐2357

##### Traditional western blotting

Protein was extracted as described above. Proteins (30 μg) were then resolved by SDS‐polyacrylamide gel electrophoresis (Bio‐Rad) and transferred to a nitrocellulose membrane (Bio‐Rad). Blots were blocked in PBST [PBS (Phosphate buffered saline) plus 0.05% Tween 20 (Sigma Aldrich)] containing 5% instant milk and incubated with primary antibody in PBST overnight at 4°Celsius. Proteins recognized by the antibody were detected by SuperSignal™ West Pico PLUS Chemiluminescent Substrate (Thermo Fisher Scientific) using a horseradish peroxidase‐coupled secondary antibody according to the manufactures’ protocol. Housekeeping genes were used to determine relative protein expression. For antibodies used: see Table 3.

#### 2.1.13 Animal experiments

C57BL/6 mice were purchased from Envigo. Mice were injected subcutaneously on the flank with 1 million LLC cells. Nu/Nu nude mice were purchased from Envigo. Mice were injected subcutaneously on the flank with 3 million H1650 cells. Mice began treatment when tumors became palpable (2‐3 mm diameter). Mice were randomized and treated every day with vehicle control, 50 mg/kg lapatinib, 20 mg/kg ketoconazole, or a combination of both until collection. Drugs were injected intraperitoneally. Drugs were dissolved in 0.1% Tween 20 with 5% DMSO in water. Tumors were collected when controls reached about 10 mm × 10 mm. Animals were housed in the University of South Florida comparative medicine facility at the Morsani College of Medicine and all protocols were reviewed and approved by the USF institutional animal care and use committee.

#### 2.1.14 Statistics

Experiments have been repeated at least twice. When comparing just two groups, such as DT vs parental or treated vs control, statistical significance for each experiment was determined using a paired *t*‐test for paired data, an unpaired t‐test for unpaired data, or a Mann‐Whitney test when the data were not normally distributed. When comparing multiple groups, statistical significance for each experiment was determined using Analysis of variance (ANOVA), the Tukey post hoc test when comparing the mean of each group with the mean of every other group, the Bonferroni post hoc test when comparing means of preselected groups, and a Kruskal‐Wallis test and the Dunn's post hoc test when the data were not normally distributed. *P < .05, **P < .01, ***P < .001, ****P < .0001. Calculations were performed and graphs were plotted using Prism 6.0 software (GraphPad). Graphs of results show the mean and error bars depict the mean plus or minus the standard error of the mean (SEM).

## 3 RESULTS

### 3.1 Establishment of EGFR TKI‐tolerant cell lines

**Figure 1:**
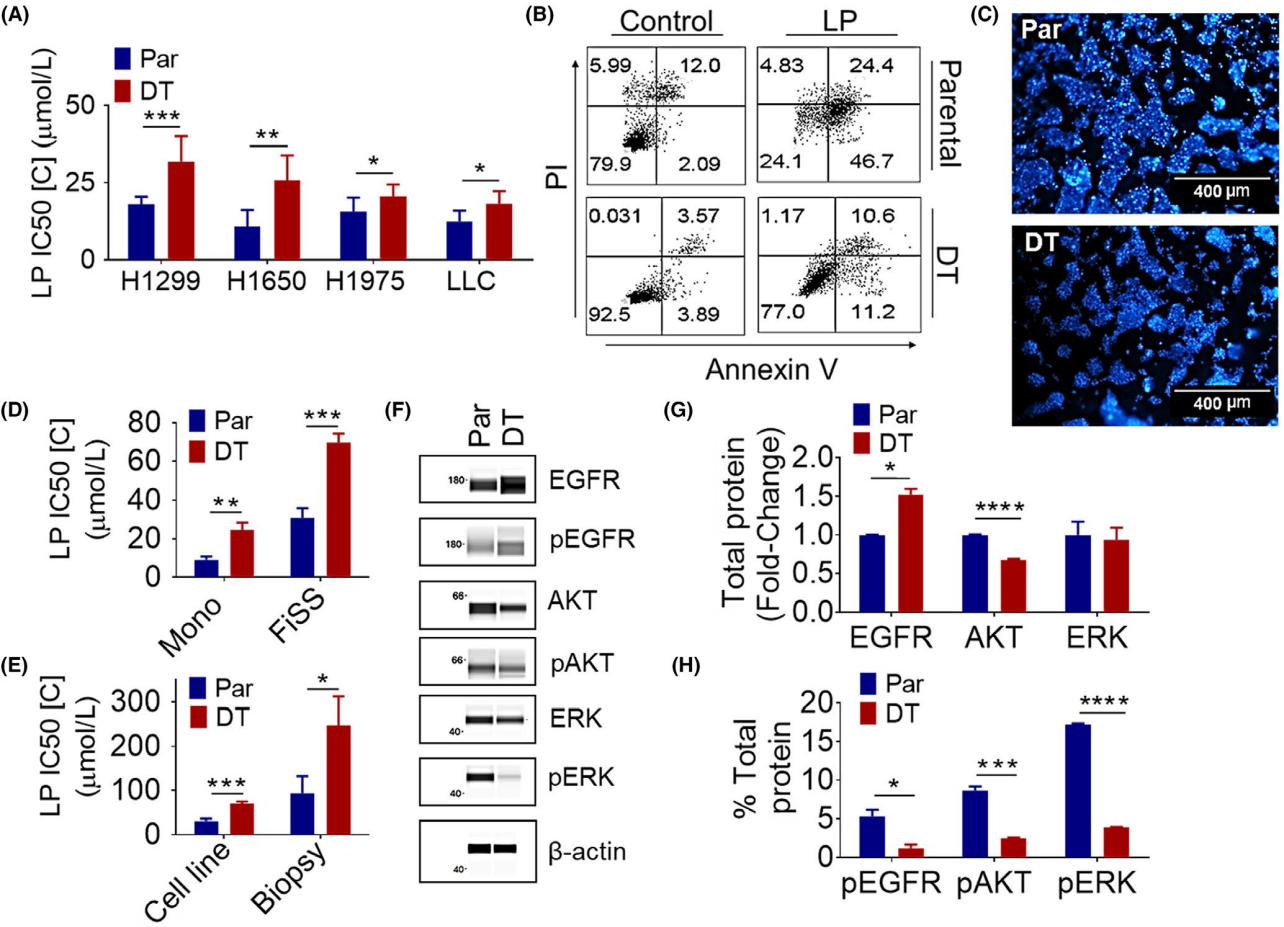
Establishment and Characterization of Acquired Lapatinib Tolerant Cell Lines. A, Lapatinib sensitivity in parental (Par) and lapatinib DT cell lines. H1650 (N = 6), H1299 (N = 8), H1975 (N = 6), and LLC (N = 3) cells were treated the day after seeding with varying concentrations of the lapatinib for 48 h. Average IC50 value ± SEM is shown. A paired *t*‐test was used to determine the significance. B, Annexin V/PI staining of parental and lapatinib DT H1975 cells after exposure to lapatinib (LP). Cells were treated the day after seeding with 20μM of lapatinib or vehicle for 48 h (N = 2). C, H1650 cell line grown on the FiSS (100X). D, A comparison of drug sensitivity in parental and lapatinib DT H1650 cell lines cultured on monolayer vs. FiSS. Monolayer drug sensitivity experiments were performed as described above. For FiSS drug sensitivity experiments cells were treated on day 5 after seeding, in triplicates, with varying concentrations of the identified drug for 48 h. Average IC50 value ± SEM in drug treated cultures on monolayer (N = 5) and the FiSS (N = 5) is shown. An unpaired *t*‐test was used to determine the significance. E, Lapatinib sensitivity in H1650 cell line vs tumor derived biopsy cultures on the FiSS. Drug sensitivity experiments were performed as described previously for the FiSS. Average IC50 value ± SEM in drug treated cultures on the FiSS (N = 5) and biopsy cultures on the FiSS (N = 4) is shown. An unpaired *t*‐test was used to determine the siignificance. F, EGFR pathway signaling protein expression in H1650 parental and lapatinib DT cells. WES automated capillary western blotting of whole cell lysate using antibodies against the indicated proteins. G‐H, The expression of proteins was determined using area under the curve (AUC) measurements generated using Compass software for Simple Western (Protein Simple). G, Average normalized fold change ±SEM of EGFR (N = 2), AKT (N = 3), and ERK (N = 3) in DT compared to parental, is shown. β‐actin was used as a loading control to normalize AUC measurements. An unpaired *t*‐test was used to determine the significance. H, Average % of normalized total protein ±SEM expression of pEGFR (N = 2), pAKT (N = 3), and pERK (N = 3) is shown. Each phospho‐protein was graphed as a percent of the respective total protein. An unpaired *t*‐test was used to determine the significance. *P < .05, **P < .01, ***P < .001, ****P < .0001

To investigate drug resistance to EGFR TKIs, we have developed lapatinib‐ and gefitinib‐tolerant lung cancer cell lines as models for de novo drug resistance. A comparison of drug sensitivity using dose response curves showed that H1299‐ and H1650‐DT cells were >2‐fold more tolerant to lapatinib than parental cells, whereas H1975‐ and LLC‐DT cells were still significantly more tolerant than parental cells, but less than 2‐fold (Figure 1A). Using an annexin V apoptosis assay we found that after exposure to lapatinib the percentage of annexin V+/PI‐ and annexin V+/PI+ cells increased more in H1975‐parental cells compared to the ‐DT cells (Figure 1B). This same trend was seen when H1299‐parental and ‐DT cells were exposed to lapatinib (Figure S1A). We also found that H1299 gefitinib‐DT cells were significantly more tolerant to gefitinib compared to parental cells, but less than 2‐fold (Figure S1B). These results suggest that 20‐60‐day exposure to EGFR TKIs can confer a tolerance to the drugs when compared to unexposed cells.

Two‐dimensional (2D) cultures are severely limited in their ability to mimic the three‐dimensional (3D) environment of the tumor microenvironment. We have developed a FiSS culture environment that creates a more representative model of tumor growth and drug resistance, which resemble in vivo tumors.^35^, ^36^, ^37^, ^38^ We found that the FiSS platform promotes the growth of 3D tumor‐like organoids (tumoroids) (Figure 1C). Using H1650 cells grown on the FiSS culture we observed about a 2‐fold greater lapatinib‐tolerance in DT cells compared to parental cells, however, the tolerance to lapatinib was increased in both cell types when cultured on the FiSS compared to monolayer (Figure 1D). We are also able to use the FiSS to grow tumor biopsies. These tumor biopsies contain not only the cancer cells, but also the stromal cells which are known to modulate the acquisition of drug resistance.^35^ We observed about a 2.5‐fold greater  lapatinib tolerance in FiSS‐tumoroids derived from DT‐H1650 tumor biopsies  compared to parental‐H1650 tumor biopsies (Figure 1E). In addition, tolerance to lapatinib increased in both H1650‐parental and ‐DT cells when cells were cultured from tumor biopsies on the FiSS as compared to the cell lines alone cultured on the FiSS (Figure 1E). These results suggest that EGFR TKI‐tolerance can be enhanced by the FiSS and further increased when tumor biopsies are cultured on FiSS providing a more accurate model of in vivo response.

### 3.2 Characterization of EGFR TKI‐tolerant cell lines

We next used the capillary electrophoresis and western blotting system, WES (Protein Simple), to determine the relative levels of EGFR, phospho‐EGFR, AKT, phospho‐AKT, ERK, and phospho‐ERK (Figure 1F). Overall, EGFR pathway downstream signaling was shown to be downregulated, with total EGFR protein increased, but a decrease seen in total AKT protein, as well as the phospho‐ AKT, ERK, and EGFR proteins in H1650‐DT cells compared to ‐parental (Figure 1G and 1H). The electropherograms obtained from WES used to quantify these data are contained in Figure S2A‐J. Seeing no upregulation in EGFR signaling that could be responsible for the increase in EGFR TKI tolerance, we then conducted mass spectrometry to find alterations in the proteome of parental vs DT cells. We found over 500 different proteins that are significantly differentially expressed in DT cells compared to parental cultures. Table 4 lists top protein candidates that were upregulated in the DT cells compared to the parental cells. Cytochrome P450 CYP51A1, which is directly involved in cholesterol synthesis, was upregulated in DT cells compared to the parental cells.^39^ The protein 14‐3‐3 (YWHAH), which is a scaffolding protein known to function in a wide variety of cellular processes was also upregulated in DT cells.^40^, ^41^, ^42^ We found that carnitine palmitoyltransferase (CPT1A), a mitochondrial enzyme that facilitates lipid catabolism was significantly upregulated in the H1975‐DT cells.^43^, ^44^ Also upregulated in the DT cells was the enzyme, sterol O‐acyltransferase 1 (SOAT1), a key enzyme converting endoplasmic reticulum cholesterol to cholesterol esters, allowing for storage in lipid droplets.^45^

**Table 4:** Selected proteins upregulated in lapatinib DT H1975 lung cancer cells compared to the parental cells

Altered proteins	Fold change	Mann‐Whitney test (*P*‐value)
CYP51A1	2.11	.021
YWHAH	1.99	.00088
CPT1A	1.39	.021
MIC60	1.30	<.0001
SOAT1	2.09	.021

### 3.3 Analysis of cholesterol metabolism in EGFR TKI‐tolerant cell lines

**Figure 2:**
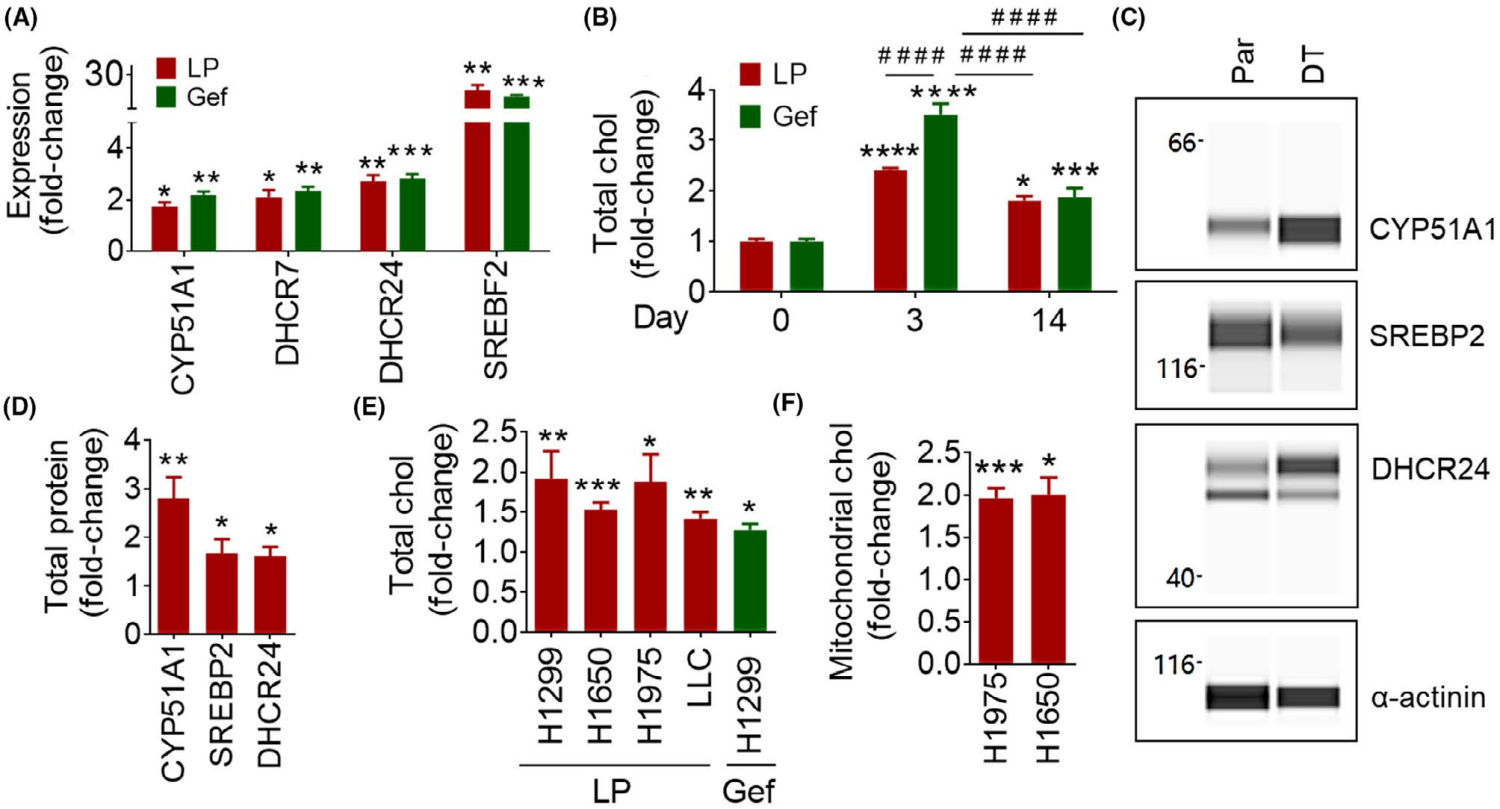
Upregulation of Cholesterol Metabolism in EGFR TKI Tolerant Cells. A, Gene expression in EGFR TKI treated parental cells. H1299 (N = 3) cells were treated at seeding with 7.5 μM lapatinib (LP) or 20 μM gefitinib (Gef) for 14 d. Average fold change ± SEM, compared to untreated, is shown. An unpaired *t*‐test was used to determine significance. B, Cholesterol (chol) content of EGFR TKI treated parental cells. H1299 cells were treated at seeding with 7.5 μM lapatinib or 15 μM gefitinib for the indicated number of days. Average fold change ±SEM, compared to untreated, is shown [Parental (N = 10), LP Day 3 (N = 6), LP day 14 (N = 2), Gef Day 3 (N = 6), Gef Day 14 (N = 4)]. * refers to comparison to day 0. # refers to comparison between two groups connected by the line. ANOVA and the Tukey post hoc test were used to determine the significance. C, Cholesterol synthesis enzyme protein expression in H1975 parental (Par) and lapatinib DT cells. WES automated capillary western blotting of whole cell lysate using antibodies against the indicated proteins is shown. D, The expression of CYP51A1 (N = 3), DHCR24 (N = 3), and SREBP2 (N = 3) was determined using AUC measurements generated using Compass software for Simple Western (Protein Simple). α‐ actinin was used as a loading control to normalize AUC measurements. Average normalized fold change ± SEM, compared to parental, is shown. An unpaired *t*‐test was used to determine the significance. E, Cholesterol content of lapatinib DT and gefitinib DT cells. Average fold change ±SEM, compared to parental, is shown [H1299 (N = 10), H1299 DT (N = 6), H1650 (N = 6), H1650 DT (N = 6), H1975 (N = 14), H1975 DT (N = 14), LLC (N = 4), LLC DT (N = 4), H1299 (N = 8), H1299 Gef DT (N = 6)]. An unpaired *t*‐test was used to determine the significance F, Mitochondrial cholesterol  content of lapatinib DT cells. Average fold change ±SEM, compared to parental, is shown [H1975 (N = 4), H1975 DT (N = 4), H1650 (N = 2), H1650 DT (N = 2)]. An unpaired *t*‐test was used to determine the significance. *P < .05, **P < .01, ***P < .001, ****P < .0001, ^####^ *P*< .0001

Three of the enzymes found to be upregulated in DT cells are directly involved in lipid and cholesterol catabolism, so we next examined whether exposure to an EGFR TKI would lead to an increase in total cellular cholesterol. We first looked at enzymes involved in cholesterol synthesis, such as CYP51A1, DHCR7 (7‐dehydrocholesterol reductase), DHCR24 (24‐dehydrocholesterol reductase), and LSS (Lanosterol synthase), as well as the transcription factor SREBF2 (Sterol regulatory element‐binding protein 2) to determine if their expression was upregulated after exposure to an EGFR TKI. After 14 days of exposure to lapatinib or gefitinib, H1299 cells showed an increase in mRNA, over untreated cells, of at least 2‐fold in CYP51A1, DHCR7, and DHCR24, as well as ~ 20‐fold increase in SREBF2 (Figure 2A). To determine if upregulation of these enzymes is leading to an increase in cellular cholesterol, H1299 cells were again exposed to 7.5μM of lapatinib or 15μM of gefitinib for up to 14 days. Total cellular cholesterol was found to be increased over untreated cells, about 2‐fold in H1299‐parental cells treated with lapatinib, and about 3‐fold when treated with gefitinib for 3 days, with cholesterol levels declining to about 1.5‐fold higher than untreated by 14 days of exposure to either TKI (Figure 2B).

We then examined mRNA transcript and protein levels of CYP51A1, DHCR7, DHCR24, LSS, and SREBF2, in DT vs parental cells. All the cell lines examined showed between a 2.5‐ and 5‐fold increase in these genes, besides H1975, which also showed about a 7.5‐fold increase in DHCR24 (Figure S3A). H1299 Gefitinib‐DT showed about a 12.5‐fold increase in SREBF2 (Figure S3A). We then used the WES system to examine protein levels of cholesterol synthesis enzymes in H1975‐parental and lapatinib‐DT cells (Figure 2C). We found a 2‐fold increase in CYP51A1, a 1.5‐fold increase in DHCR24 and LSS, and a 1.25‐fold increase in SREBP2 protein levels in lapatinib‐DT cells compared to parental (Figure 2D). The electropherograms obtained from WES used to quantify these data are contained in Figure S3B‐G. As with parental cells exposed to EGFR TKIs, H1299, H1650, H1975, and LLC lapatinib‐DT cells showed an upregulation of cholesterol 1.5‐2‐fold, compared to their parental cells (Figure 2E). We also found an increase in total cellular cholesterol in H1299 gefitinib‐DT cells of about 1.5‐fold compared to parental cells (Figure 2E). These results show an increase in total cellular cholesterol levels, as well as enzymes directly involved in cholesterol synthesis in both parental cells treated with EGFR TKIs and EGFR TKI‐DT cells.

We next looked at how increased levels of cholesterol could contribute to increased EGFR TKI‐tolerance. A comparison of lipid raft staining of parental‐ and DT‐H1975 cells with 48 hour lapatinib treatment showed no statistical difference between the groups (Figure S4A‐B). Since we have already shown that the EGFR pathway is downregulated in H1650 lapatinib‐DT cells and there is no increase in lipid raft signaling caused by increased cholesterol, which would theoretically function to turn on the EGFR pathway in DT cells, we do not think that the elevated cholesterol is contributing to an increase in lipid raft formation and leading to EGFR TKI‐tolerance in our DT model. To determine whether an increase in mitochondrial cholesterol could contribute to increased EGFR‐TKI tolerance, we then isolated mitochondrial protein from parental and DT cells; mitochondrial isolation was verified using COX4 as a mitochondrial marker and vinculin as a cytoplasmic marker (Figure S5A‐C). We found that in both H1650‐ and H1975‐DT cells mitochondrial cholesterol was upregulated about 2‐fold compared to parental (Figure 2F).

### 3.4 EGFR TKI and ketoconazole combination therapy inhibits the development of resistance

**Figure 3:**
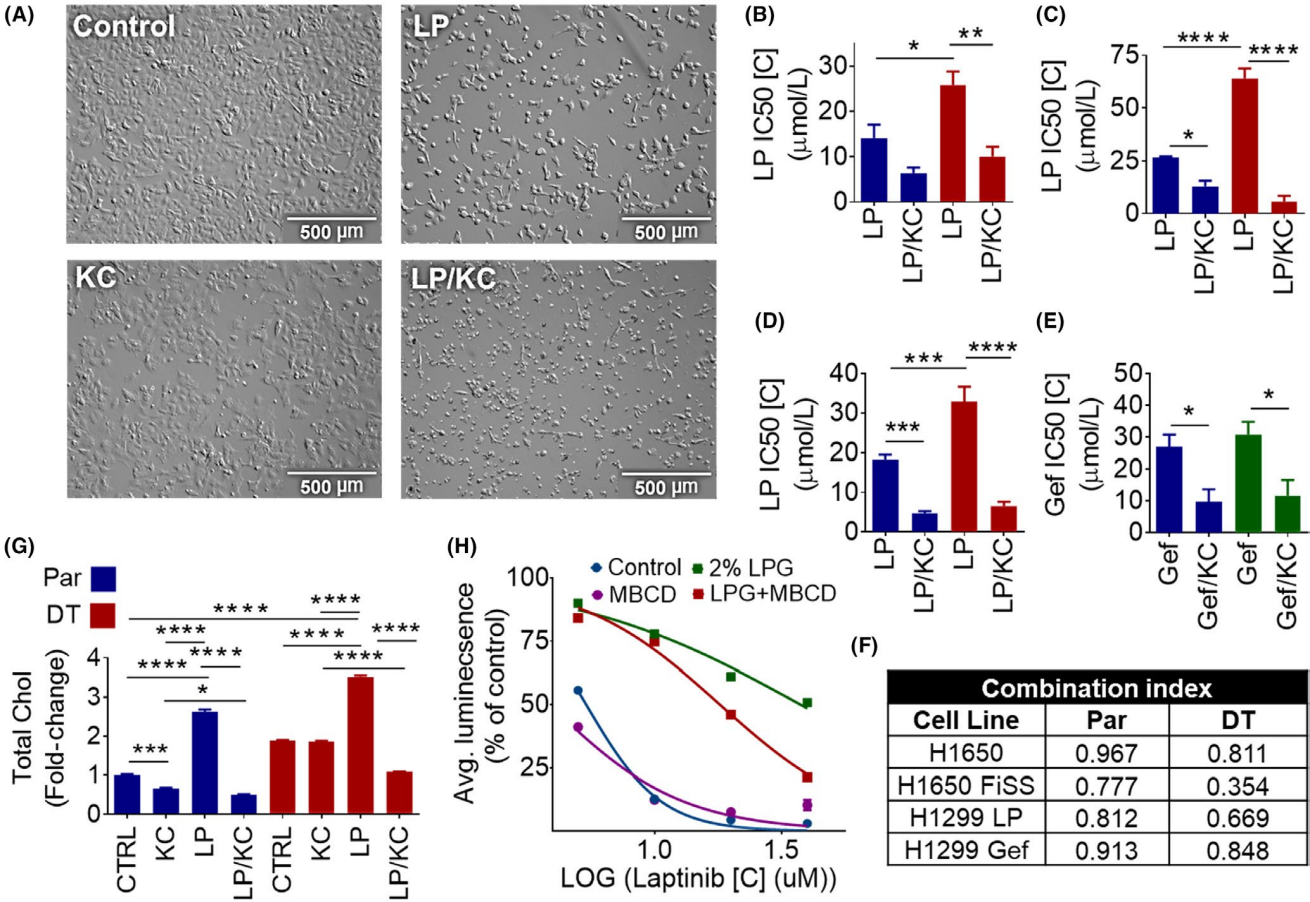
Ketoconazole and EGFR TKI Therapy Overcomes EGFR TKI Tolerance by Stopping Upregulation of Cholesterol. A, Cellular morphology after lapatinib and ketoconazole combination therapy. Parental and lapatinib DT H1650 cells were plated and treated with 12.5 μM lapatinib (LP), 15μM ketoconazole (KC), or a combination of both. They were allowed to grow for 72 h. Bright field microscopy at 100X is shown. B‐E, Lapatinib and ketoconazole combination sensitivity in parental and DT cell lines. Cells were treated the next day after plating with varying concentrations of the identified drug/drugs for 48 h. Monolayer and FiSS drug sensitivity experiments were performed as described previously. Average IC50 value ± SEM on monolayer and the FiSS is shown, H1650 (N = 4) (B) H1650 FiSS (N = 3) (C), H1299 lapatinib (LP) (N = 5) (D), and H1299 gefitinib (Gef) (N = 5) (E). ANOVA and the Bonferroni post hoc test were used to determine the significance. F, CI was calculated for each cell line using the average IC50 values. G, Cholesterol content after lapatinib and ketoconazole combination therapy. Parental (Par) and lapatinib DT H1650 cells were treated the day after seeding with 10 μM lapatinib, 20 μM ketoconazole, or a combination of both for 48 h. Average fold change ±SEM, compared to untreated parental, is shown (N = 2). ANOVA and the Tukey post hoc test were used to determine the significance. H, Lapatinib sensitivity in H1650 parental cells. H1650 (N = 3) cells were plated in complete growth media or complete growth media containing 2% Lipogro (LPG). Cells were treated the day after seeding, in triplicates, with varying concentrations of the lapatinib for 48 h. Dose response curve is shown. *P < .05, **P < .01, ***P < .001, ****P < .0001

After finding upregulation of CYP51A1, leading to increased cellular cholesterol levels in EGFR TKI‐treated cells, we next set out to test the potential of a CYP51A1 inhibitor, ketoconazole, used in combination with EGFR TKIs to overcome the development of EGFR TKI‐tolerance.^46^ Addition of 20μM ketoconazole to the culture media of H1650‐parental cells, along with 6μM lapatinib, reduced the viability of cells (Figure 3A). Ketoconazole alone at this dose was not very toxic to the cells (Figure S6A). In all of the lapatinib‐DT cell lines, the addition of ketoconazole lowered the IC50 of lapatinib in DT cells more when compared to parental [Figure 3B (H1650), 3C (H1650 FiSS), and 3D (H1299)]. Ketoconazole was also able to lower the IC50 of gefitinib in H1299‐parental and gefitinib‐DT cells (Figure 3E). We next used the Combination Index (CI) calculation to determine the level of synergism between EGFR TKIs and ketoconazole.^21^ H1650‐parental and ‐DT cells treated with lapatinib and ketoconazole, both on monolayer and FiSS, showed a CI < 1, which indicates that the combination acts synergistically (Figure 3F). H1299‐parental, as well as lapatinib‐ and gefitinib‐DT cells, also showed synergistic response between the EGFR TKIs and ketoconazole, with their CI < 1 (Figure 3F). These data show the synergistic potential of ketoconazole and EGFR TKI combination therapy in lung cancer cells.

We next investigated the effects of lapatinib and ketoconazole combination therapy on cellular cholesterol levels. Ketoconazole treatment alone did not significantly decrease cellular cholesterol levels in all cell lines tested (Figures 3G and S6A). In both H1650‐ and LLC‐parental and ‐DT cells we found that the addition of ketoconazole to lapatinib treatment was able to halt the upregulation of cholesterol levels seen after treatment with lapatinib alone [Figure 3G (H1650) and S6B (LLC)]. Lastly, we examined the effect of incubating H1650 cells with excess amounts of cholesterol during exposure to lapatinib. Cholesterol is transported in a bound complex with protein as a low‐density lipoprotein (LDL) to cells throughout the body, where it is then taken into cells and packaged into lysosomes and separated from the LDL protein complex into free cholesterol, which is then transported to the cell membrane and membrane–bound organelles.^22^, ^23^ Thus, to get parental cells to take up excess cholesterol from the cell culture media, we added 2% Lipogro Bovine Cholesterol Concentrate to complete growth media at the time of cell seeding for IC50 experiments. This caused the lapatinib tolerance of parental cells to increase about 7‐fold, from 5.4 uM to 34 uM, compared to cells grown in complete growth media. However, when the cholesterol depleting agent, methyl‐β‐cyclodextrin (MBCD), was added to the media in addition to the Lipogro, the tolerance to lapatinib was lowered by 2‐fold to 18uM (Figure 3H). These results show that increased levels of cholesterol in the cells can lead to increased EGFR TKI‐tolerance, which can be reversed by stopping cholesterol production or depleting cholesterol levels.

### 3.5 Induction of apoptosis by the combination therapy

**Figure 4:**
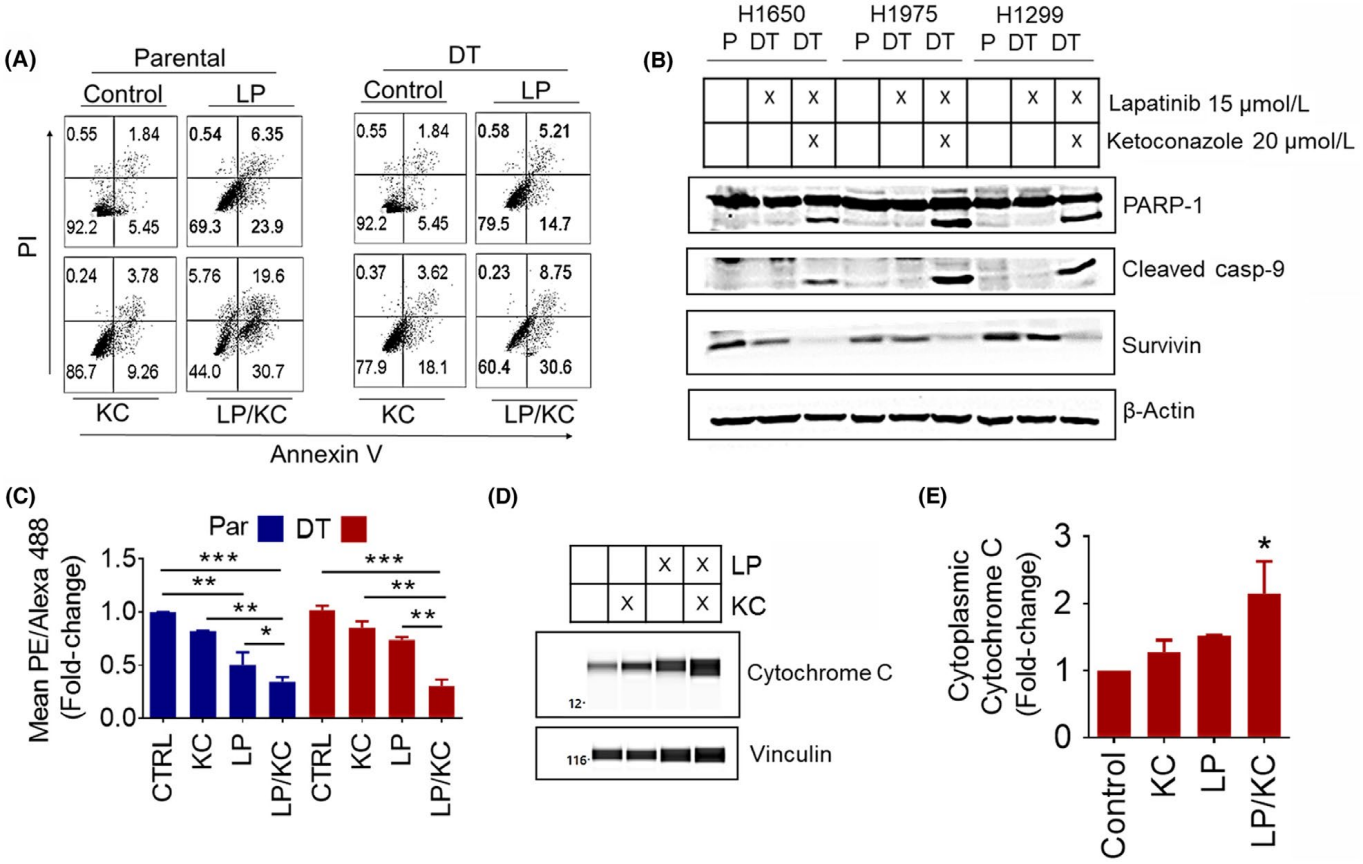
Lapatinib and Ketoconazole Therapy Induces Apoptosis Through Mitochondrial Membrane Depolarization. A, Parental (Par) and DT cells were treated the day after seeding with 15 μM lapatinib (LP), 20 μM ketoconazole (KC), or a combination of both for 48 h (N = 2). A, Annexin V/PI staining of parental and lapatinib DT H1650 cells. B, Apoptosis proteins in parental and lapatinib DT cells. The expression of the indicated proteins was determined using traditional western blotting. β‐Actin was used as a loading control. C, JC‐1 staining of parental and lapatinib DT H1299 cells. Average PE/Alexa 488 ratio ±SEM is shown. ANOVA and the Tukey post hoc test were used to determine the significance. D, Cytochrome C release in lapatinib DT H1299 cells after exposure to lapatinib, ketoconazole or combination. WES automated capillary western blotting of whole cell lysate using antibodies against the indicated proteins. E, Analysis of cytosolic cytochrome C was determined using area AUC measurements generated using Compass software for Simple Western (Protein Simple). Vinculin was used as a loading control to normalize AUC measurements. Average normalized fold change ±SEM, compared to untreated, is shown. A Kruskal‐Wallis and the Dunn's post hoc tests were used to determine the significance. *P < .05, **P < .01, ***P < .001

Next, we determined whether the combination of lapatinib and ketoconazole induced better cell death than either treatment alone. Using annexin V and PI staining we showed that in H1650‐parental and ‐DT cells the combination of 15μM lapatinib and 20μM ketoconazole was able to increase the levels of both annexin V positive and annexin V/PI double positive cells over either drug by itself after 48 hours exposure (Figure 4A). This same trend was seen in H1975‐parental and ‐DT cells (Figure S6C). Using traditional western blotting, we found downregulation of survivin, and cleavage of both caspase‐9 and PARP (Poly ADP ribose polymerase) in H1299, H1650, and H1975 cells after treatment with the combination therapy, whereas lapatinib alone was unable to cause these effects (Figure 4B). These data suggest that both parental and DT cells are undergoing apoptosis after treatment with a combination of lapatinib and ketoconazole.

Due to the fact that we saw an increase in mitochondrial cholesterol levels in EGFR TKI cells, we next evaluated the effect of the combination therapy on mitochondrial membrane potential. To measure this we used JC‐1 dye, which exhibits potential‐dependent aggregation in the mitochondria and a red to green color shift caused by changes in the concentration of red fluorescent JC‐1 aggregates, as mitochondrial depolarization is indicated by a decrease in red emitting aggregated JC‐1 and an increase in green emitting soluble JC‐1.^47^ We found a nearly 75% decrease in mitochondrial membrane potential in H1299‐parental and ‐DT cells after 48 hours of combination therapy (15 μM lapatinib and 20 μM ketoconazole) (Figure 4C).  However, only in parental cells did lapatinib alone cause a significant decrease, of about 50% compared to control, in mitochondrial membrane potential (Figure 4C). Depolarization of the mitochondrial membrane can lead to the release of cytochrome C from the mitochondria to the cytosol in cells undergoing apoptosis.^27^ Using the WES system, we were able to show that after treatment with 15 μM lapatinib and 20 μM ketoconazole combination therapy for 48 hours, H1299 lapatinib‐DT cells had a significant increase in cytochrome C levels in their cytosol compared to untreated, indicating release of cytochrome C into the cytosol (Figure 4D,E). The electropherograms obtained from WES used to quantify this data are contained in Figure S7A‐D. Taken together, we found an inhibition of EGFR TKI‐induced cholesterol upregulation and an activation of mitochondrial apoptosis after treatment with the EGFR TKI plus ketoconazole combination therapy .

### 3.6 Mouse model of acquired EGFR TKI resistance

**Figure 5:**
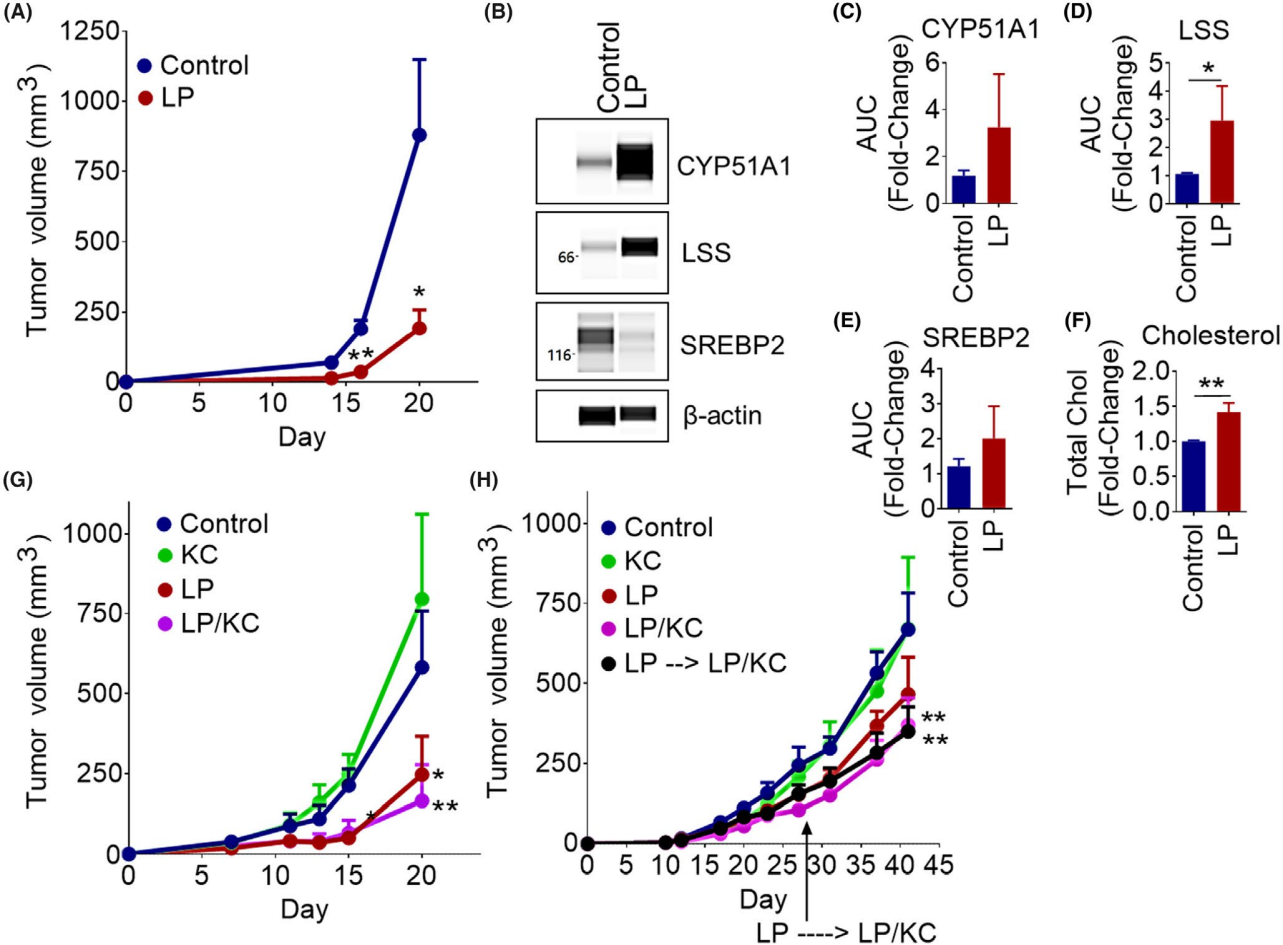
Lapatinib and Ketoconazole Combination Therapy Overcomes EGFR TKI Resistance in an in Vivo Mouse Model of NSCLC. A, Treatment of LLC1 tumors with lapatinib. C57BL/6 mice were inoculated with LLC1 cells. Tumors were treated every day from day 6 to day 13 with vehicle control (N = 2) or 50mg/kg lapatinib (LP) (N = 4). Tumor volume ±SEM over time is shown. An unpaired *t*‐test was used to determine the significance. B, Cholesterol synthesis enzyme protein expression in vehicle and lapatinib (LP) treated LLC1 tumors. WES automated capillary western blotting of whole tumor cell lysate using antibodies against the indicated proteins. C‐E, The expression of CYP51A1 (N = 6) (C), LSS (N = 6) (D), and SREBP2 (N = 3) (E) was determined using AUC measurements generated using Compass software for Simple Western (Protein Simple). β‐actin was used as a loading control to normalize AUC measurements. Average normalized fold change ±SEM, compared to vehicle treated tumors, is shown. A Mann‐Whitney test was used to determine the significance. F, Cholesterol content of vehicle and lapatinib (LP) treated LLC1 tumors. Average fold change ±SEM, compared to vehicle treated tumors, is shown (N = 7). An unpaired *t*‐test was used to determine the significance. G‐H, Mice were inoculated with tumor cells. Mice began treatment when tumors became palpable (2‐3mm diameter) and were treated every day with vehicle control, 50mg/kg lapatinib (LP), 20mg/kg ketoconazole (KC), or a combination. Tumor volume ±SEM over time is shown. G, C57BL/6 mice (N = 5/group) inoculated with LLC1 cells and treated. ANOVA and the Bonferroni post hoc test were used to determine the significance. H, Nu/Nu nude mice (N = 5/group) were inoculated with H1650 cells. One group of mice that had been receiving lapatinib treatment was switched to the lapatinib and ketoconazole combination therapy at day 28. ANOVA and the Bonferroni post hoc test were used to determine the significance. *P < .05, **P < .01

We then set out to develop a mouse model of acquired EGFR TKI‐DT to test our novel combination therapy. Using an immunocompetent LLC allograft mouse model, we were able to replicate the in vitro model of EGFR TKI‐DT. Lapatinib treatment was able to decrease tumor size at least threefold compared to control, however the lapatinib treated tumors steadily grew after treatment was withdrawn at day 13 (Figure 5A). We next collected the tumors and examined both total cellular cholesterol as well as cholesterol synthesis enzymes. Using the WES capillary immunoassay system, we examined the levels of the cholesterol synthesis enzymes CYP51A1, LSS, and DHCR24 in both control and lapatinib treated LLC mouse tumors (Figure 5B). The electropherograms obtained from WES used to quantify this data are contained in Figure S8A‐H. We found a threefold increase in CYP51A1and LSS, and a twofold increase in SREBP2 protein levels in lapatinib treated tumors compared to control tumors [Figure 5C (CYP51A1), 5D (LSS), and 5E (SREBP2)]. In these tumors we also found cholesterol levels within the lapatinib treated group to be 1.5‐fold higher than control tumors (Figure 5F). These data show that the upregulation of cholesterol synthesis after treatment with an EGFR TKI also occurs in an in vivo mouse model as seen in vitro.

Lastly, we used an in vivo tumor model to determine the effectiveness of our novel combination therapy. In LLC tumors we found that the combination therapy significantly inhibted tumor growth better than lapatinib alone (Figure 5G). In H1650 tumors however, we found that only the combination was able to decrease tumor size by about twofold compared to control at day 41 (Figure 5H). Even switching from lapatinib treatment alone to the combination therapy at day 26 was able to decrease tumor size by about twofold compared to control at day 41 (Figure 5H). These data highlight the ability of ketoconazole and EGFR TKI combination therapy to overcome the development of EGFR TKI resistance in vivo.

## 4 DISCUSSION

Upregulation of cholesterol synthesis in cancer has been shown to correlate with poor prognosis in patients and plays a role in the development of drug resistance, as overexpression of cholesterol pathway genes has been observed in refractory tumors.^23^, ^26^, ^48^ Total cellular cholesterol was shown to be upregulated in gefitinib resistant cells.^20^ In this study, cell lines with different sensitivities to gefitinib were used as a model of resistance. The cells were not under exposure to gefitinib when cholesterol was isolated, but rather they were comparing levels between the different cell lines. We provide evidence for the first time that upregulation of cholesterol synthesis is a direct response to EGFR TKI exposure. We also show that mitochondrial cholesterol levels are upregulated in lung cancer cells exposed to EGFR TKIs. Moreover, by blocking mitochondrial cholesterol buildup using ketoconazole, we were able to overcome the development of EGFR‐TKI tolerance in lung cancer cells. EGFR TKIs have been shown to mediate their apoptotic effects via inhibition of Akt/Erk signaling, which downregulates anti‐apoptotic Bcl‐2 family proteins that are responsible for binding and inhibiting Bax and Bak, which once free, can cause MOMP and activate apoptotic cell death.^18^, ^20^, ^27^, ^28^ Several studies have shown that elevated levels of mitochondrial cholesterol in cancer cells may be able to protect cancer cells against mitochondrial apoptosis by reducing the ability of Bax to insert into the mitochondrial membrane thus impairing MOMP and the release of cytochrome c.^29^, ^30^, ^31^ To our knowledge, no previous studies have linked this phenomenon to EGFR TKI exposure and resistance.

By using our models of acquired EGFR TKI resistance we were able to study the acquisition of resistance to EGFR TKIs in lung cancer cells and develop a combination therapy with the potential to overcome EGFR TKI resistance. After treatment with EGFR TKI plus ketoconazole combination therapy we found a reversal of the EGFR TKI induced cholesterol upregulation and an activation of apoptosis. Without the buildup of cholesterol in the mitochondrial membrane the cells were not able to resist MOMP. Thus, apoptosis was activated by mitochondrial membrane depolarization caused by MOMP, leading to cytochrome c release into the cytosol and subsequent cleavage of caspase‐9 and PARP, leading to cell death in both parental and EGFR TKI‐DT cells.

Ketoconazole, a potent inhibitor of CYP51A1 along with CYP3A4, is an FDA approved broad‐spectrum systemic antifungal agent and also used in the treatment of the hormone‐dependent prostate cancer, due to its ability to block steroidogenesis.^46^, ^49^, ^50^ Ketoconazole has also been studied to examine its cytotoxic effects when used as a single agent, and synergistic when combined with chemotherapeutics.^49^, ^50^, ^51^ When it comes to ketoconazole and EGFR TKIs, the available data are limited. In one study, the authors examined the impact of ketoconazole induced CYP3A4 inhibition on lapatinib pharmacokinetics.^51^ They found that systemic exposure to lapatinib was significantly increased by inhibition of CYP3A4, most likely due to the fact that CYP3A4 is the primary metabolizing enzyme for lapatinib.^51^ CYP3A4 is the most abundant cytochrome P‐450 expressed in human liver and it contributes to the metabolism of many drugs in use today. This may serve to at least partially explain the combination effects seen by some drugs with ketoconazole, a potent CYP3A4 inhibitor.^52^ We have shown that through the inhibition of CYP51A1, ketoconazole was able to halt the buildup of cholesterol in the mitochondrial membrane, resulting in the cells not being able to resist MOMP in the presence of an EGFR TKI. To our knowledge ketoconazole is not currently being used as a treatment for any types of lung cancer.

**Figure 6:**
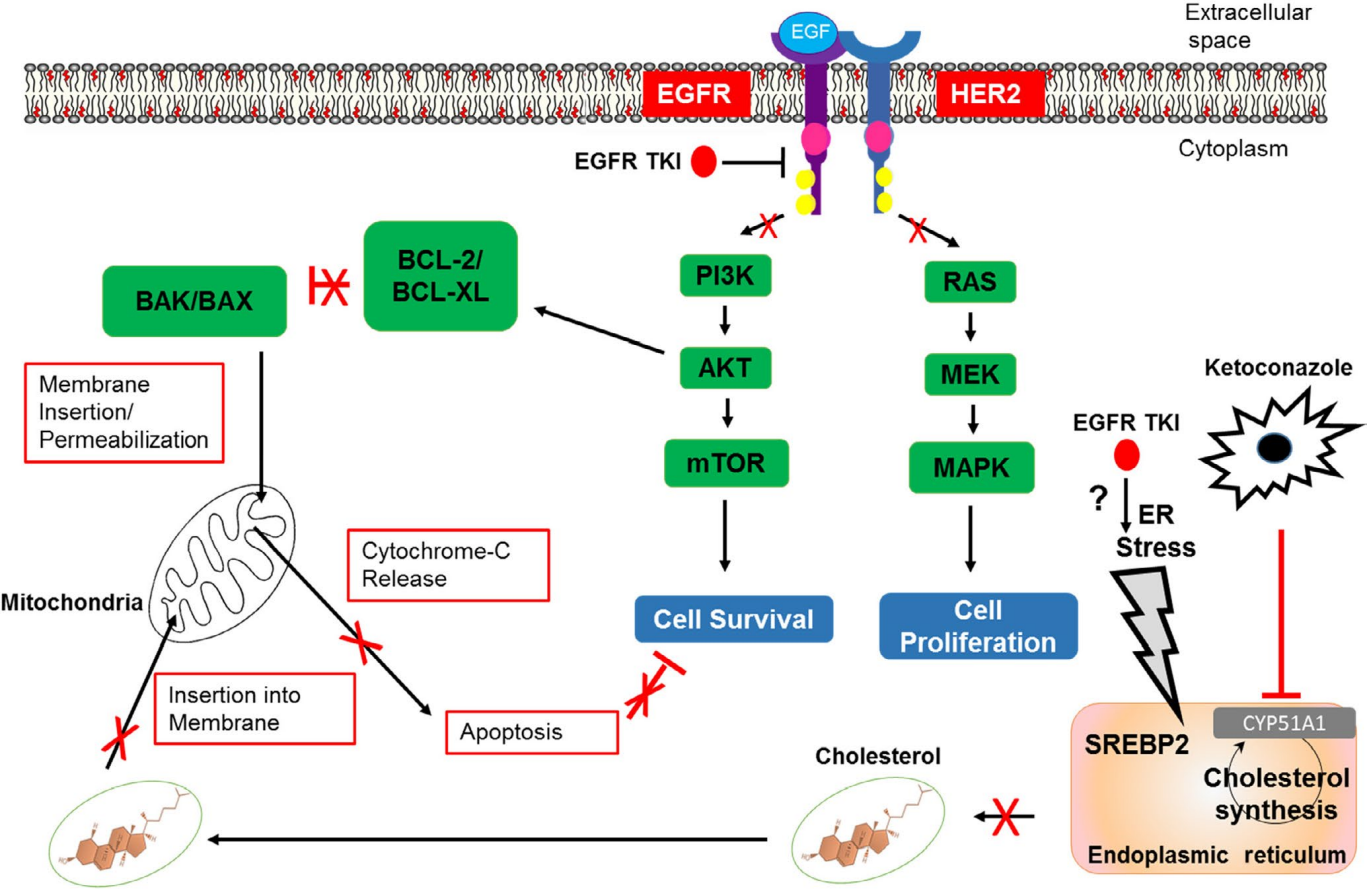
Mechanism of Action. Upregulation of cholesterol is caused by induction of ER stress after EGFR TKI exposure. This is inhibited by ketoconazole not allowing the cell to increase cholesterol levels within the mitochondrial membrane. Without this survival mechanism, EGFR TKI exposure leads to MOMP, release of cytochrome C, and apoptosis

One last question remaining is how is treatment with EGFR TKIs is causing upregulation of cholesterol. In our model we found upregulation of SREBP2, which is a transcription factor that controls the transcription of cholesterol synthesis genes.^53^ It has been shown that ER stress induces activation of SREBP2 and a subsequent cholesterol accumulation in cancer cell lines.^54^ EGFR TKI‐treatment has been linked to the induction of ER stress response and this is one of the reasons that patients experience diarrhea as a common side effect of EGFR TKIs.^55^, ^56^ Another study found that erlotinib‐induced ER stress signaling can promote the survival of EGFR TKI‐persister cells after exposure to erlotinib through transcriptional adaptation via an epigenetic state change.^56^ Taken together, these studies highlight the possibility that EGFR TKI induced ER stress is causing activation of SREBP2 and subsequent activation of cholesterol synthesis (Figure 6). EGFR TKI exposure leadin to ER stress dependant induction of SREBP2 would require further testing in our model. The knowledge gained in our studies could be useful in the future development of therapies to overcome acquired resistance to EGFR TKIs, a major problem in patients receiving EGFR TKIs.

## 5 CONCLUSIONS

In conclusion, we have created and validated a model for EGFR TKI‐resistance, successfully used this model to examine the mechanisms of cell survival following EGFR TKI‐exposure, and elucidated a method of overcoming this survival to eliminate development of EGFR TKI‐resistance. We showed that cellular and mitochondrial cholesterol synthesis is upregulated after exposure to EGFR TKIs. We also demonstrated the effectiveness of a ketoconazole plus EGFR TKI combination therapy in overcoming this phenomenon. The knowledge gained in this study could be useful in the future development of therapies to overcome acquired resistance to EGFR TKIs.

## CONFLICT OF INTEREST

The authors declare no potential conflicts of interest.

## AUTHOR CONTRIBUTIONS

Mark C. Howell, Shyam Mohapatra, and Subhra Mohapatra designed research; Mark C. Howell, Ryan Green, Elspeth Foran, Rajesh Nair, Stanley Stevens analyzed data; Mark C. Howell, Ryan Green, Roukiah Khalil, Elspeth Foran, Waise Quarni, Aleksandr Grinchuk, and Andrew Hanna performed research**;** Mark Howell, Ryan Green, Shyam Mohapatra, and Subhra Mohapatra wrote the paper.
