# Supplementary material for: Lung cancer cells survive epidermal growth factor receptor tyrosine kinase inhibitor exposure through upregulation of cholesterol synthesis
Source: FASEB Bioadv. 2019 Dec 28;2(2):90–105. doi: 10.1096/fba.2019-00081 (PMC7003654; doi:10.1096/fba.2019-00081)
Supplement: Supplementary file 1 [file FBA2-2-90-s001.pdf]

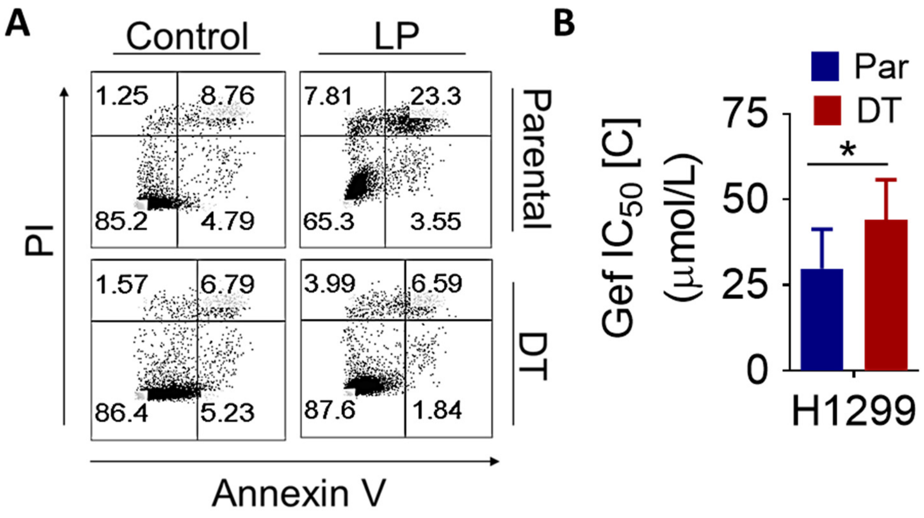

SFigure 1: Characterization of EGFR TKI Tolerant Cell Lines

A) Annexin V/PI staining of parental and DT H1299 cells after exposure to lapatinib. Cells were treated the day after seeding with 20µM of lapatinib or vehicle for 48 hours (N=2). **B)** Gefitinib sensitivity in parental and gefitinib DT cell lines. H1299 (N=5) cells were treated the day after seeding, in duplicates, with varying concentrations of the gefitinib for 48 hours. Average IC_{50} value ±SEM is shown. Paired T-Test was used to determine significance. * = $p<0.05$

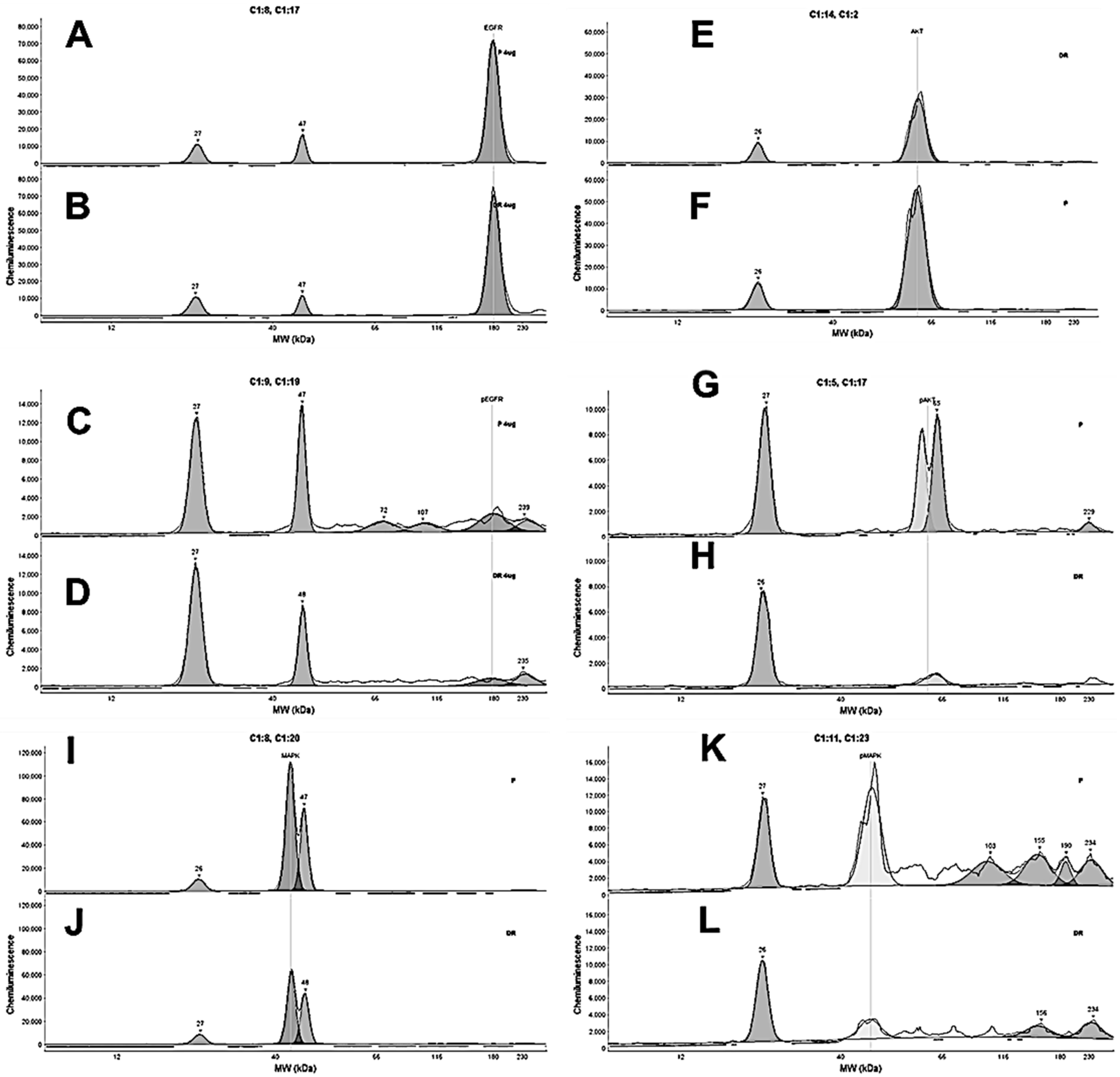

SFigure 2: Electropherograms from WES Data in Figure 1F

A-J) Electropherograms data from H1650 P and lapatinib DT. WES automated capillary western blotting of whole cell lysate using antibodies against the indicated proteins [(A= Par EGFR/β-actin) (B= DT EGFR/β-actin) (C= Par pEGFR) (D= DT pEGFR) (E= Par AKT) (F= DT AKT) (G= Par pAKT) (H= DT pAKT) (I= Par ERK) (J= DT ERK) (K= Par pERK) (L= DT pERK)].

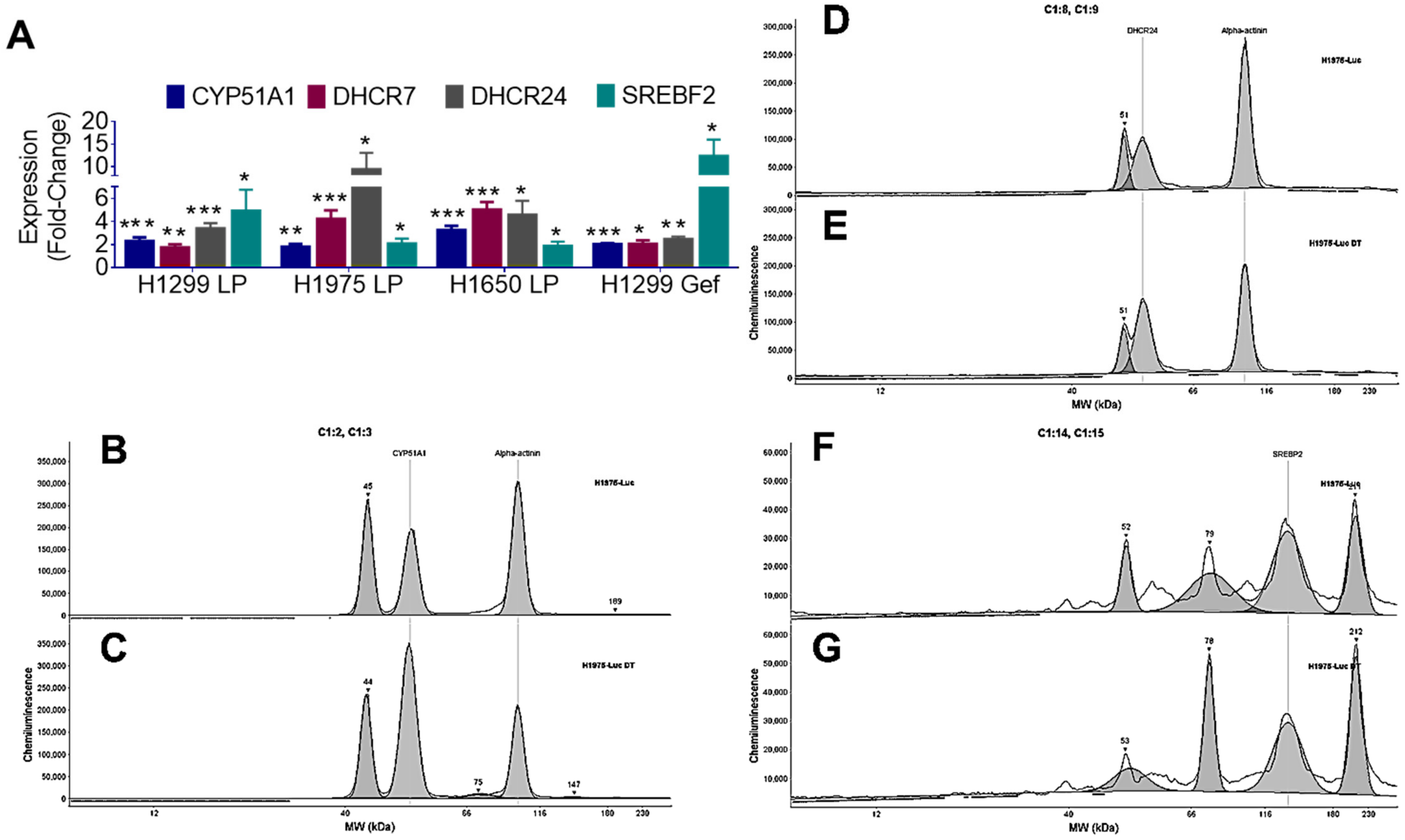

SFigure 3: Cholesterol Synthesis in Parental vs DT Cells

A) Gene expression in EGFR TKI DT cells. Average fold change ±SEM, compared to parental, is shown [H1299 LP (N=9), H1975 LP (CYP51A1 N=6) (DHCR7 N=6) (DHCR24 N=9) (SREBP2 N=9), H1650 LP (N=6), H1299 Gef (N=3)]. Unpaired T-Test was used to determine significance. **B-G)** Electropherograms data from H1975 P and lapatinib DT. WES automated capillary western blotting of whole cell lysate using antibodies against the indicated proteins [(B= Par CYP51A1/α-actin) (C= DT CYP51A1/α-actin) (D= Par DHCR24/α-actin) (E= DT DHCR24/α-actin) (F= Par SREBP2) (G= DT SREBP2)]. * = $p<0.05$, ** = $p<0.01$, *** = $P<0.001$

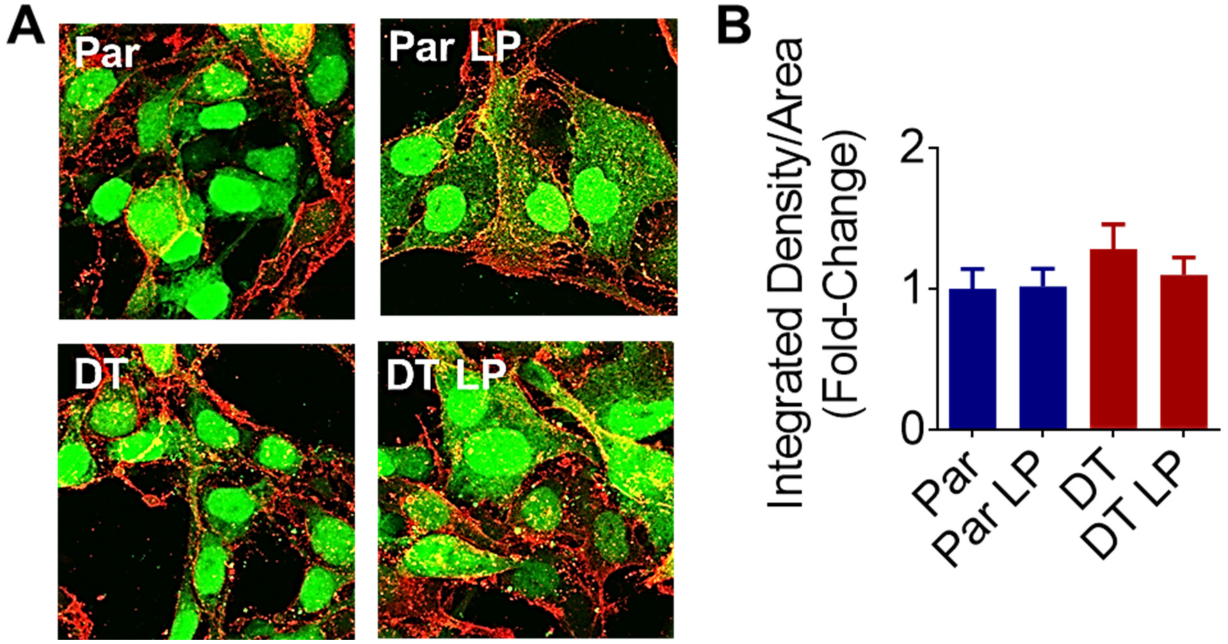

SFigure 4: Lipid Rafts Analysis in Parental vs DT Cells After Lapatinib Exposure

A) Lipid rafts staining in H1975 parental vs DT cells. Parental and lapatinib DT H1975 cells were plated and treated with 7.5M lapatinib 24 hours after plating. They were allowed to grow for 48 more hours. Max projections of the confocal Z-stack images (600X) are shown. **B)** Quantification of lipid raft staining in H1975 parental vs DT cells. Z-stack images were analyzed for fluorescence intensity per area using ImageJ software (NIH). Graph shows fold change compared to parental. ANOVA and the Tukey post hoc test was used to determine significance.

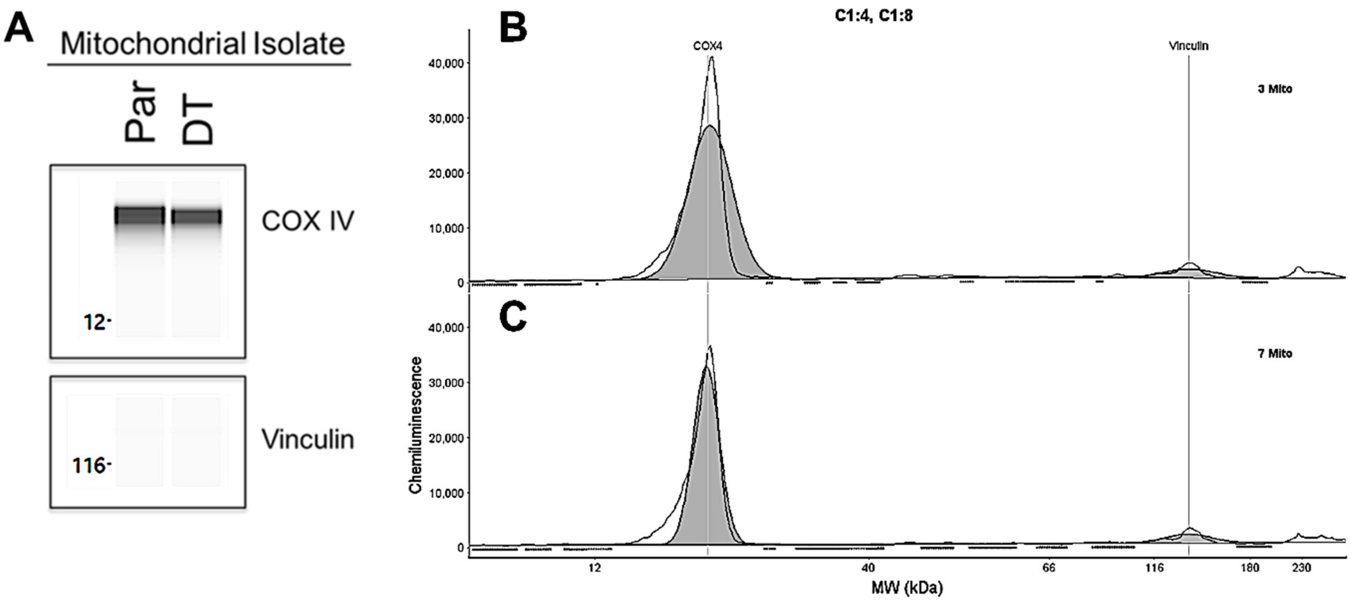

SFigure 5: Mitochondrial Isolation

A) Mitochondrial isolation in H1975 parental and lapatinib DT cells. WES automated capillary western blotting of mitochondrial isolate using antibodies against the indicated proteins. **B-C)** Electropherograms data of isolated mitochondria from H1299 P and lapatinib DT. WES automated capillary western blotting of whole cell lysate using antibodies against the indicated proteins [(B= Par COX4/Vinculin) (C= DT COX4/Vinculin)].

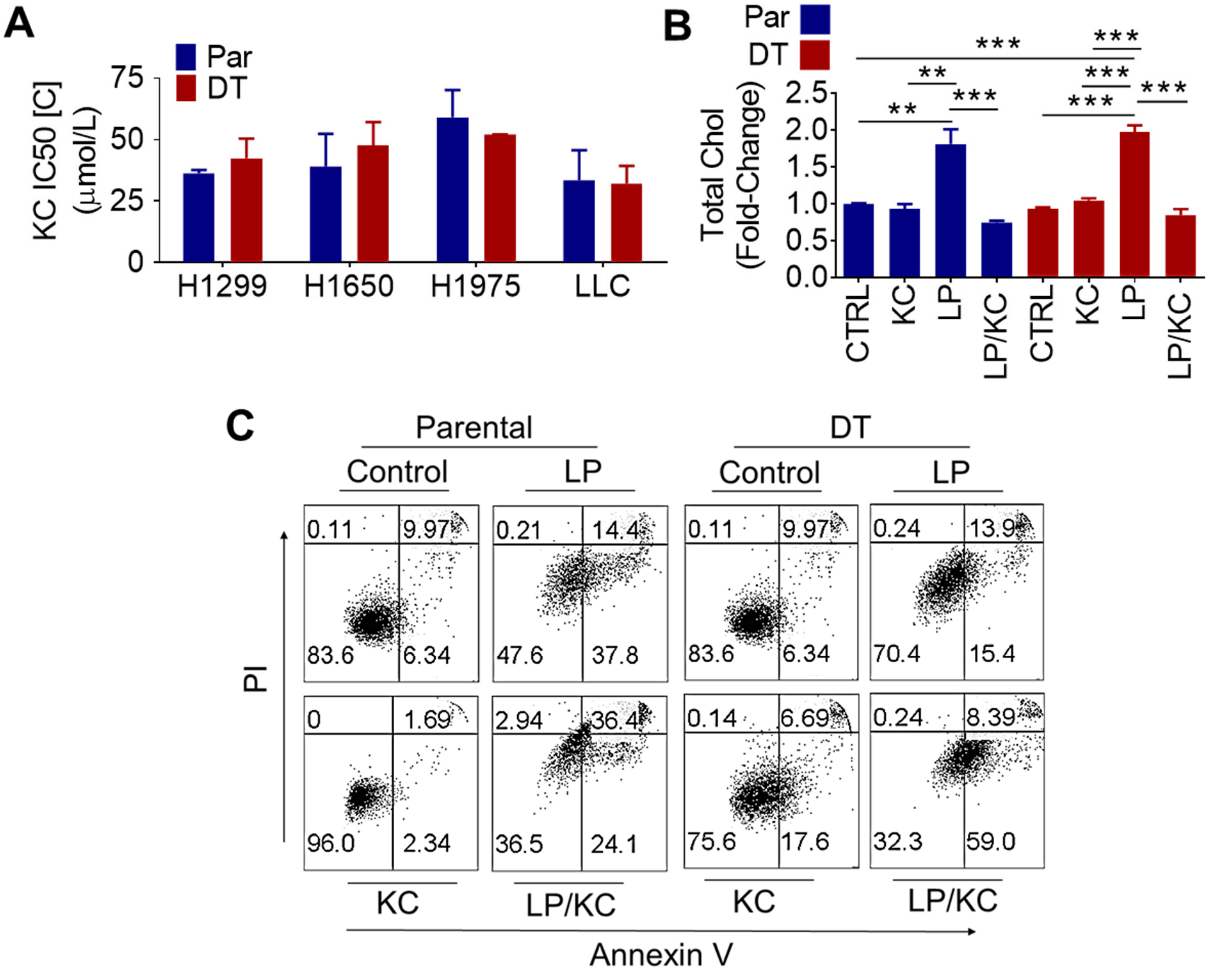

SFigure 6: Lapatinib and Ketoconazole Combination Therapy is Able to Halt the Upregulation of Cholesterol Synthesis and Induce Apoptosis

A) Ketoconazole sensitivity in parental and lapatinib DT cell lines. . H1650 (N=2), H1299 (N=2), H1975 (N=2), and LLC (N=3) cells were treated the day after seeding, in duplicates, with varying concentrations of the gefitinib for 48 hours. Average IC50 value ±SEM is shown. Paired T-Test was used to determine significance. **B)** Cholesterol content after lapatinib and ketoconazole combination therapy. Parental and lapatinib DT LLC cells were treated the day after seeding with 10μM lapatinib, 20μM ketoconazole, or a combination of both for 48 hours. Average cholesterol content ±SEM as a percentage of untreated parental culture is shown (N=2). ANOVA and the Tukey post hoc test was used to determine significance. **C)** Annexin V/PI staining of parental and lapatinib DT cells after exposure to lapatinib and ketoconazole combination therapy. Parental and DT H1975 cells were treated the day after seeding with 15μM lapatinib, 20μM ketoconazole, or a combination of both for 48 hours (N=2). * = p<0.05, ** = p<0.01, *** = P<0.001

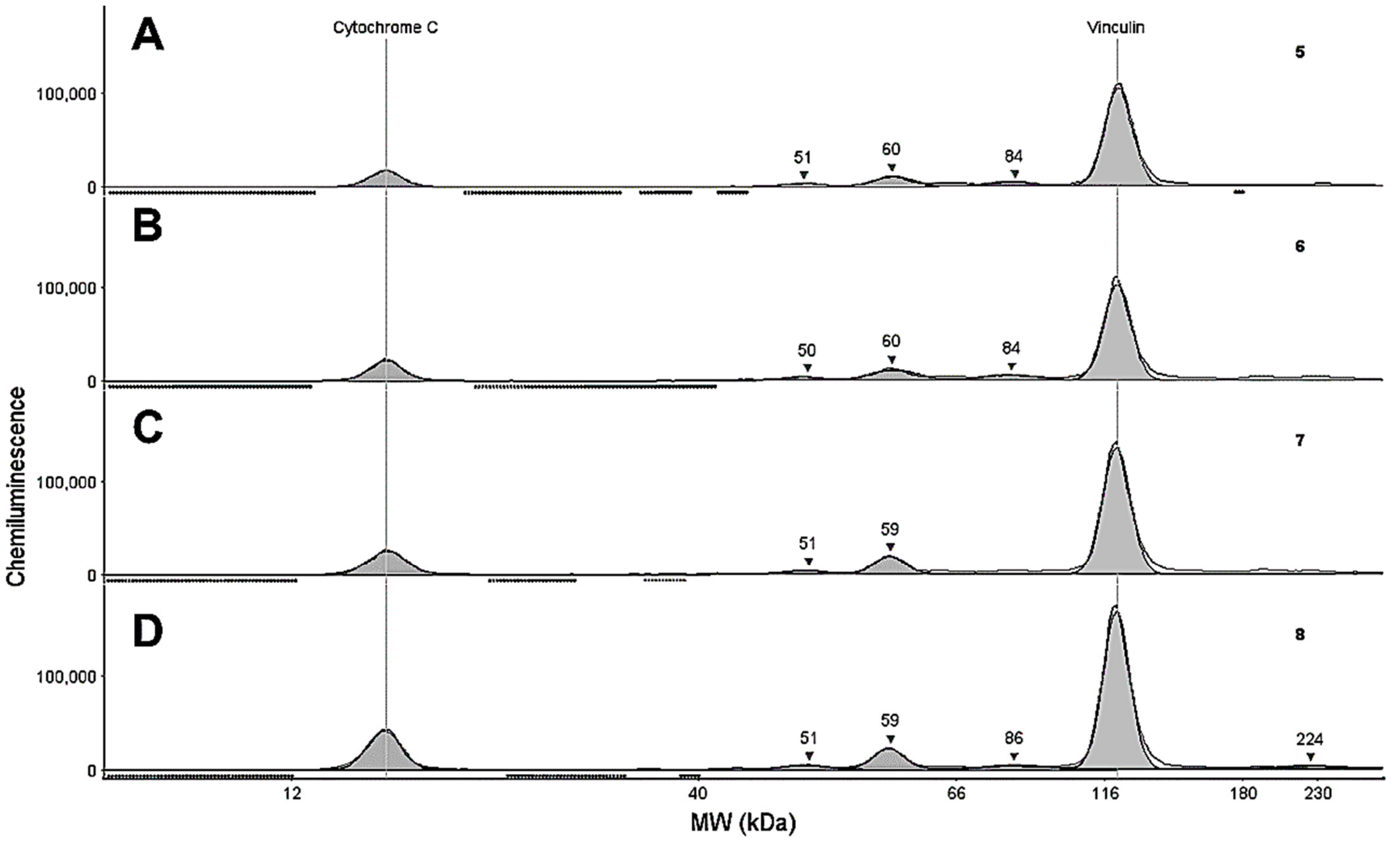

SFigure 7: Electropherograms from WES Data in Figure 4D

A-D) Electropherograms data of cytoplasmic extracts from H1299 lapatinib DT. WES automated capillary western blotting of whole cell lysate using antibodies against the indicated proteins [(A= Control Cytochrome C/Vinculin) (B= KC Cytochrome C/Vinculin) (C= LP Cytochrome C/Vinculin) (D= LP/KC Cytochrome C/Vinculin)].

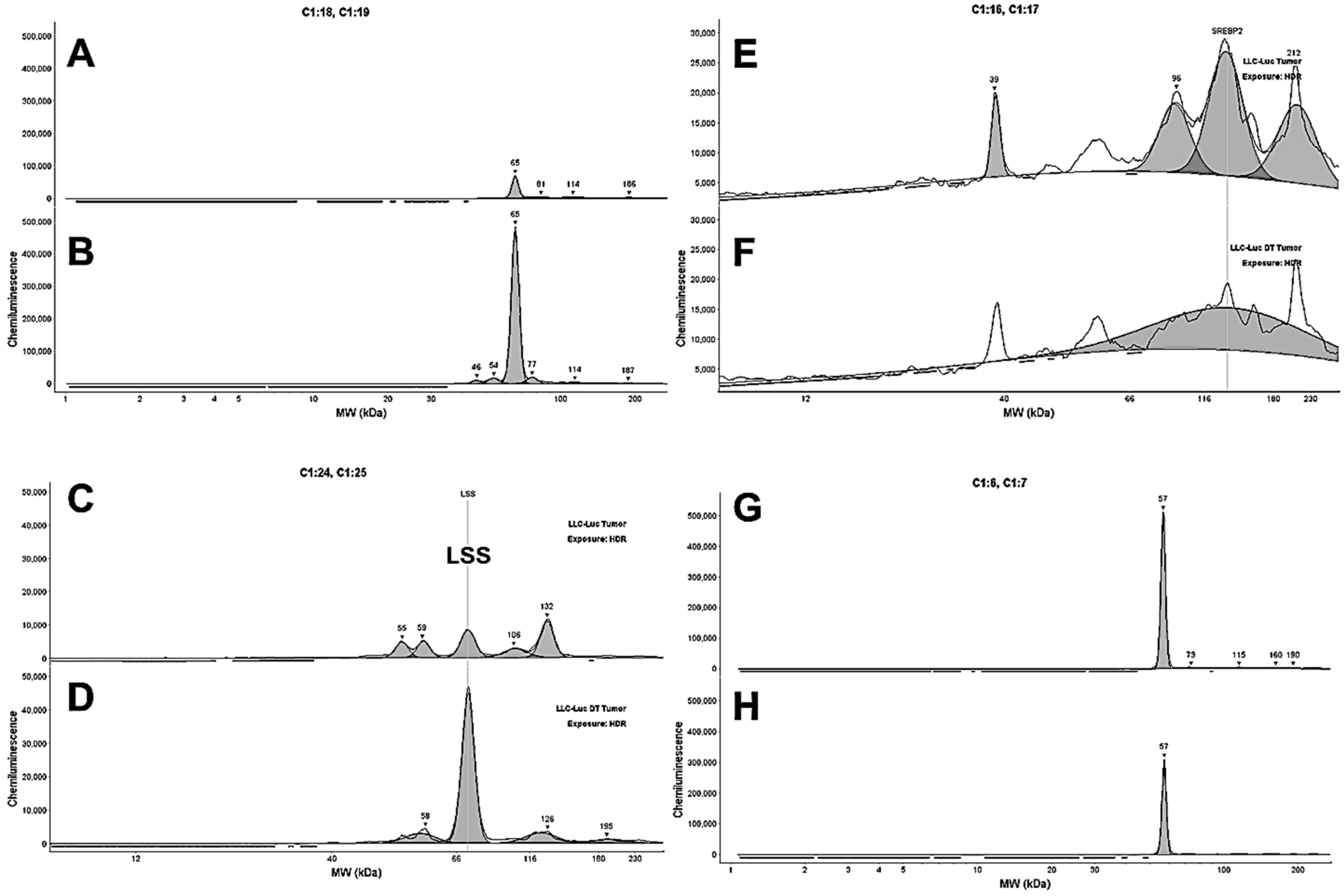

SFigure 8: Electropherograms from WES Data in Figure 5B

A-H) Electropherograms data from control and LP treated LLC tumor extracts. WES automated capillary western blotting of whole cell lysate using antibodies against the indicated proteins [(A= Control CYP51A1) (B= LP CYP51A1) (C= Control LSS) (D= LP LSS) (E= Control SREBP2) (F= LP SREBP2) (G= Control β-actin) (H= LP β-actin)].
